# Programmable and sustainable scaling up of squaramide-based compounds through customizable paper-based platforms

**DOI:** 10.1039/d6ra09121k

**Published:** 2026-10-20

**Authors:** Panagiota M. Kalligosfyri, Antonella Ilenia Alfano, Stefano Cinti, Margherita Brindisi

**Affiliations:** a Department of Pharmacy (DoE 2023–2027), University of Naples Federico II Via D. Montesano 49 Naples 80131 Italy antonellailenia.alfano@unina.it panagiota.kalligosfyri@unina.it; b Sbarro Institute for Cancer Research and Molecular Medicine, Center for Biotechnology, College of Science and Technology, Temple University Philadelphia Pennsylvania 19122 USA; c Bioelectronics Task Force at University of Naples Federico II Naples 80126 Italy; d Department of Chemistry, Faculty of Science, Chulalongkorn University Bangkok 10330 Thailand

## Abstract

The transition toward sustainable chemical manufacturing requires reaction systems that are scalable, energy-efficient, and inherently aligned with Green Chemistry principles. Here, we introduce the first programmable strategy for paper-based scale-up organic synthesis, revisiting the use of filter paper as predictive manufacturing platform, rather than a mere analytical substrate and an innovative support for chemical reactions. By utilizing wax-patterned paper-based platforms with tunable diameters, we establish quantitative relationships between platform's geometry, reagent loading, and product yield, enabling seamless translation from microliter screening to multi-gram synthesis. Using squaramide synthesis as a model, we demonstrate that increasing paper-based platform's size preserves reaction efficiency while maintaining outstanding green metrics, including a constant E-factor, high atom and carbon efficiencies, and excellent EcoScale scores. To further enable preparative-scale operation, large paper-based platforms are integrated into a modular 3D-printed scaffold that enhances mechanical stability and suppresses solvent evaporation. Unlike conventional scale-up strategies requiring complex flow equipment or energy-intensive infrastructure, our pump-free, cost effective and highly accessible platform exploits paper's porosity and capillary-driven transport to achieve on-demand, sustainable manufacturing. This work establishes paper as a new programmable reaction platform, offering a scalable, low-cost, and customizable solution toward decentralized and environmentally responsible chemical synthesis.

## Abstract 2

Programmable and sustainable paper-based scale up for squaramides synthesis.

## Graphical abstract

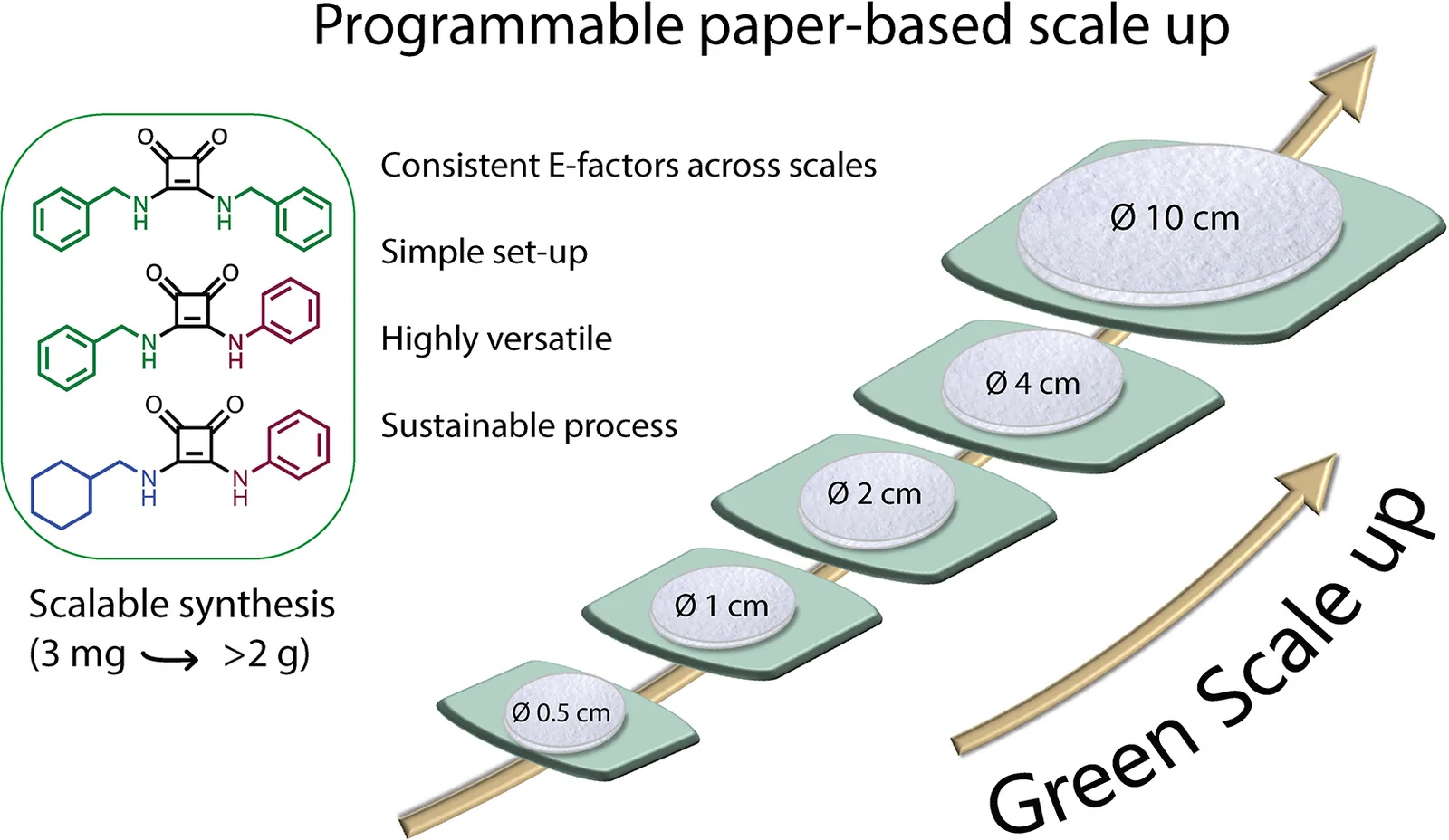

## Introduction

The modern chemical manufacturing sector, including the subsector of fine chemicals, faces an urgent imperative to transition toward decentralized, benign-by-design, and energy-efficient operational platforms that align with the 12 Principles of Green Chemistry.^1^ Conventional synthetic processes often rely on resource-intensive equipment, large solvent volumes, and materials that contribute significantly to environmental burdens, highlighting the need for more sustainable reaction platforms.^2^ Reaction scale up remains a major challenge in classic batch processing. Scaling a chemical transformation is far more complex than simply increasing reactor size and often requires extensive optimization, time, and resources. Continuous-flow chemistry is a well-established strategy for process intensification and scale-up, offering improved control over reaction conditions, heat and mass transfer, and safety.^3–6^ In photochemistry, flow reactors also mitigate Beer–Lambert limitations by enhancing light penetration, enabling efficient scale-out.^7^ However, many current systems still depend on external pumping infrastructure, non-biodegradable materials, and complex fabrication, which can limit their sustainability and accessibility.^8^ In parallel, mechanochemistry has emerged as a powerful solvent-free approach, and recent advances have enabled its implementation in both batch and continuous-flow scale-up strategies, although challenges related to homogeneous energy transfer, process reproducibility and post-transformation purification steps still need to be addressed.^9,10^

To realize the full sustainability benefits of process intensification during scale-up, manufacturing platforms must maximize atom economy, reduce solvent consumption, and minimize waste generation. This requires more efficient utilization of reactants, operation under conditions that lower overall fluid requirements, suppression of byproduct formation, reduced solvent demand during purification and filtration, and lower energy consumption during drying processes.^2,11^ Achieving these objectives requires technologies that enhance reaction performance while minimizing the material and energy footprint of the manufacturing platform.

Paper-based reaction platforms have recently attracted attention as a promising solution to the limitations of traditional chemical manufacturing. Historically, paper has been utilized primarily to purify and pretreat samples, store samples and or reagents, preconcentrate low abundant targets of interest.^12^ Paper and other cellulose threads have only rarely been exploited as an active reaction platform, such as for the synthesis of nanoparticles or the enzymatic amplification of nucleic acids.^13–15^ Indeed, our previous work represented a pioneering step in this direction, where paper substrates were exploited for the first time as a viable platform for organic synthesis.^16^ Paper-based substrates are sustainable, biodegradable, inexpensive, and naturally porous, enabling seamless filtration and spontaneous fluid transport through capillary action without the need for external pumps. These characteristics allow paper to function simultaneously as a reaction support while reducing energy consumption and material waste. Even though these structural merits have driven the widespread commercial and academic adoption of microfluidic paper-based analytical devices for low-cost assays,^12^ the potential of paper substrates to serve as macro-scale, preparative chemical reactors for organic synthesis remains heavily underexplored.

This synthetic deficit is particularly notable given the escalating demand for sustainable protocols to construct high-value molecular frameworks, such as squaramides. Characterized by their rigid four-membered cyclobutenedione substructure, squaramides possess a unique dual hydrogen-bond donor–acceptor architecture that enables the formation of highly directional hydrogen-bonding networks.^17^ Therefore, they represent important structural motifs in medicinal chemistry,^18–20^ and for their application in organic synthesis, due to their anion coordination properties,^21,22^ and their incorporation in the structure of several asymmetric organocatalysists.^23,24^

In our previous work, we addressed the ecological footprint of squaramide production by pioneering a sustainable methodology utilizing paper-based discs patterned with hydrophobic wax boundaries.^16^ This simple platform architecture enabled both faster reaction and product purification maintaining high yields and excellent green metrics. Despite these advantages, a fundamental hurdle for these paper platforms is the deeply ingrained perception of scale limitation. Paper substrates are traditionally viewed as single-use screening tools restricted entirely to micro- or milligram-scale outputs, inherently blocking their translation into viable, preparative-scale manufacturing strategies. This limitation is especially true for reactions yielding solid products, as precipitate formation can easily clog the porous structure or compromise the structural stability of the paper.

Herein, for the first time to our knowledge, we address this throughput bottleneck by presenting a programmable, geometric scaling framework for paper-based synthesis, governed by the controlled radial expansion of the reaction disc diameter. First, we investigate the correlation between the design, printing and heat treating of the wax barriers and its effect on the paper disc diameter to enable “on-demand” preparation of the paper-based platforms with high batch-to-batch reproducibility. By expanding the paper disc boundaries across a spectrum from 0.5 to 10 cm, we map out the fundamental chemical engineering relationships connecting cellulosic surface area and 3D fiber pore volume to absolute reagent loading thresholds and mass yields. Crucially, we demonstrate that tuning these physical boundaries provides predictable fluidic capacities spanning three orders of magnitude (∼11 µL to 10 mL), creating a direct, seamless bridge from microscale optimization screens to preparative multi-gram isolation. Moreover, we report excellent green metrics, including atom economy and atom efficiency, carbon and reaction mass efficiency. One of the most relevant findings is the scale-up independent behavior of E-factor in our newly conceived protocol. In addition, calculated EcoScale score, is fully in line with the excellent values of our previous work, is excellent. Finally, the integration of these scalable discs into a modular 3D-printed supporting scaffold, particularly for the larger disc sizes (2–10 cm), provides mechanical support and helps minimize solvent evaporation during operation. Our work highlights the transformative nature of paper-based synthesis and shifts the paradigm of our approach from a proof-of-concept methodology into a predictive, scalable, and customizable platform, opening new opportunities for accessible and sustainable chemical manufacturing.

## Experimental section

### Structural and geometric fidelity in relation to liquid loading capacity

The wax-printed paper-based platforms were heat-treated during fabrication, as described in the SI, and this may have caused variations in the disc dimensions. Therefore, to evaluate the precision of the fabrication process, we compared the designed diameters of the discs against the actual diameters measured after printing and subsequent thermal treatment. A consistent dimensional deviation was observed, which proved to be independent of the paper-based platform size. Specifically, the printed wax boundaries were consistently 0.5 mm smaller than the initial Autodesk Fusion design. Following thermal treatment, which induces the vertical and lateral flow of molten wax into the cellulose matrix, the effective reaction zone diameter decreased by a total of 1 mm across all studied disc diameters (0.5 to 10 cm) (Fig. 1A). This uniform “shrinkage” indicates that the lateral diffusion of wax is governed by the paper's capillary structure rather than the overall disc geometry. Because this 1 mm variance is constant regardless of size, it allows for predictable design compensations, ensuring high geometric reproducibility when transitioning from microscale to preparative-scale synthesis.

**Fig. 1:**
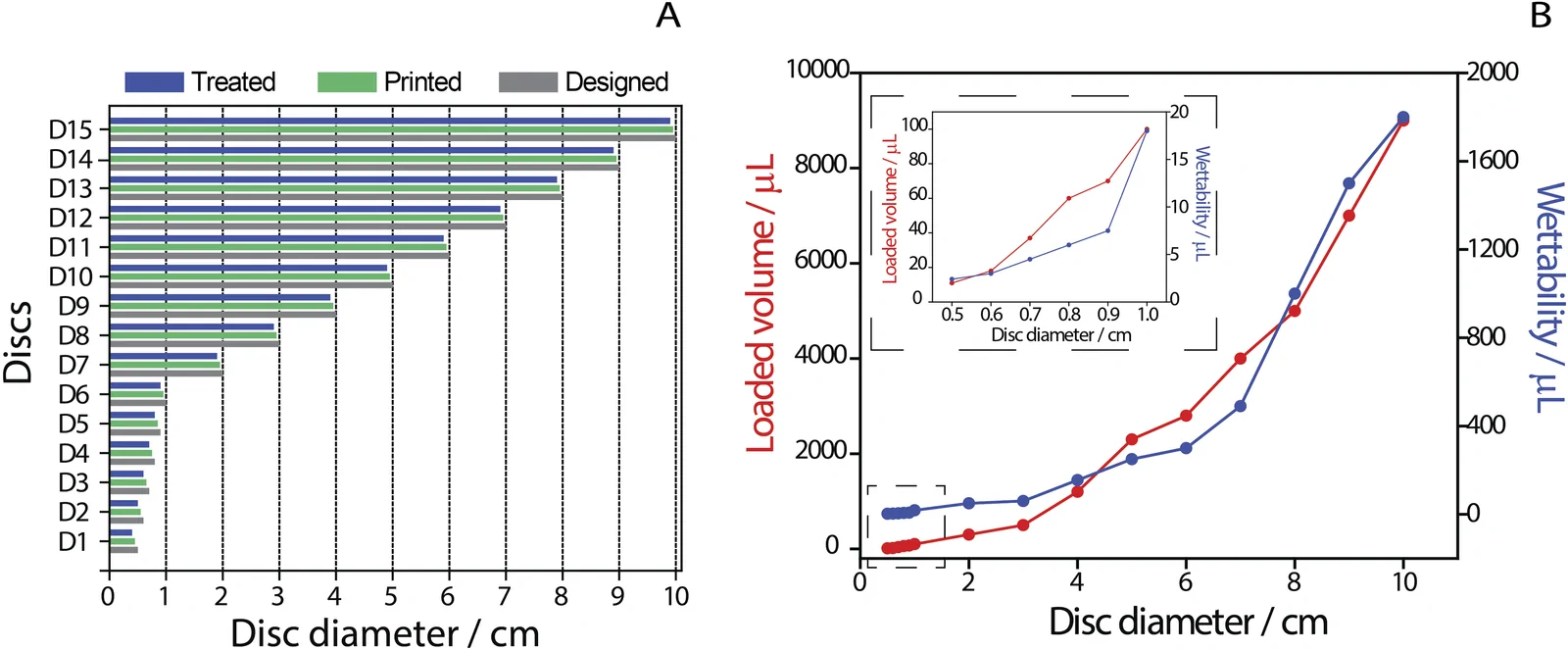
Dimensional characterization and fluidic capacity of scalable paper-based reaction platforms. (A) Comparison of disc diameters across the design, printing, and thermal treatment stages due to wax diffusion. (B) Evaluation of liquid loading capacity and the paper's wettability as a function of disc diameter. The inset provides a magnified view of the smaller paper disc dimensions (0.5, 0.6, 0.7, 0.8, 0.9 and 1 cm).

In order to thoroughly understand the correlation between the dimensions of the paper-based platform and the loaded volume, and to ensure full programmability, the relationship between disc diameter and maximum wetting and loading volumes were investigated to determine the operational limits at each scale. The wettability was experimentally calculated by adding small volumes (10 µL for up to 0.5 cm and 100 µL for up to 10 cm diameter) of a red dye solution. Here, wettability was defined as the amount of red dye solution required to completely wet the entire paper-disc surface, with the maximum value corresponding to the volume required to achieve complete wetting of the disc. The exact experimental volumes for each disc diameter are presented in Table S1. As expected, the wettability increases with disc size, with the discs accommodating volumes ranging from ∼11 µL (0.5 cm) up to ∼10 mL (10 cm). Separately, the reaction-loading volume was defined as the volume of reagent solution introduced into the wax-defined reaction zone before splitting, as determined by the advancing liquid meniscus up to the point at which the solution reached the wax boundary and extended outside the defined reaction zone. Since the loading volume increases with the diameter increase (Fig. 1B) and as expected with area increase (Fig. 2A), a more practical and predictive linear relationship was established by plotting the loading volume against the disc area (Fig. 2B). This linear correlation is defined by the equation: *y* = −7.9 + 105.6*x* (where *y* is the loading volume in µL and *x* is the disc area in cm^2^). This empirical equation serves as a calibration tool, enabling researchers to calculate the required reagent volumes based on their chosen disc dimensions. The slope of the linear correlation (105.6 µL cm^−2^) characterizes the specific absorptive capacity of the Whatman filter paper Grade 1 (thickness: 180 µm; pore size: 11 µm; 0.25 psi wet burst; speed: 150 s/100 mL) substrate. This coefficient is governed by the physical architecture of the cellulose matrix, specifically its thickness and pore-volume distribution, as well as by the experimental configuration and loading conditions, and therefore provides a practical basis for estimating reagent volumes for different disc dimensions.

**Fig. 2:**
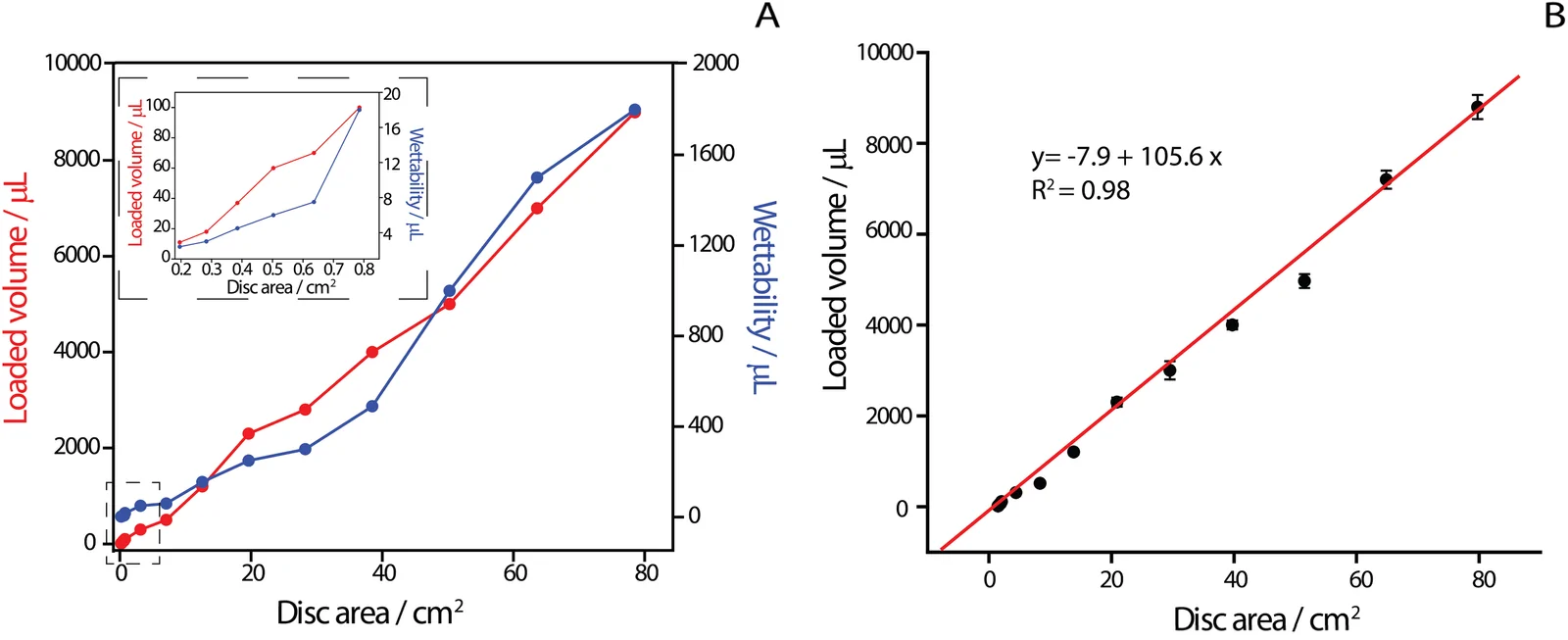
(A) Evaluation of liquid loading capacity and the paper's wettability as a function of disc area. The inset provides a magnified view of the smaller paper disc dimensions (0.5, 0.6, 0.7, 0.8, 0.9 and 1 cm). (B) Linear correlation of the loading volume against the disc area. Data are presented as mean ± standard deviation from three independent measurements (*n* = 3).

### Paper-based scale-up platforms set up

To streamline operational simplicity for large-scale reactions on the paper-based platform, specifically when employing discs with diameter ranging from 6 to 10 cm, a custom 3D-printed support system was developed. By exploiting the customizable nature of 3D printing,^25^ the support was specifically designed to match the dimensions and operational requirements of the paper-based platform, providing a simple, low-cost, and adaptable solution for large-scale reactions. While smaller paper discs (*e.g.* 0.5, 1, 4 cm) are sufficiently lightweight to be secured with adhesive tape at two contact points, the wider discs carry a significantly higher product load on a gram scale (*e.g.* for 10 cm disc ∼2 grams of product). This increased mass can compromise the mechanical stability of the paper, making it prone to sagging or tearing during handling and drying. The 3D-printed support system consists of a 12 × 12 cm rectangular frame with a height of 6 cm. The base incorporates a 5 × 5 mm grid pattern, which provides robust physical reinforcement while maintaining a high percentage of open area for efficient product drying. To further enhance stability, a detachable component was designed with slightly larger dimensions (12.6 × 12.6 cm) to fit over the base. This part features two 2 mm wide side slits and a 10 cm circular cutout to precisely house the paper-based platform (Fig. 3). In laboratory settings where a 3D-printed support is not readily available, the paper disc can alternatively be secured by applying adhesive tape along all 4 sides of the substrate to maintain the necessary stability during the reaction.

**Fig. 3:**
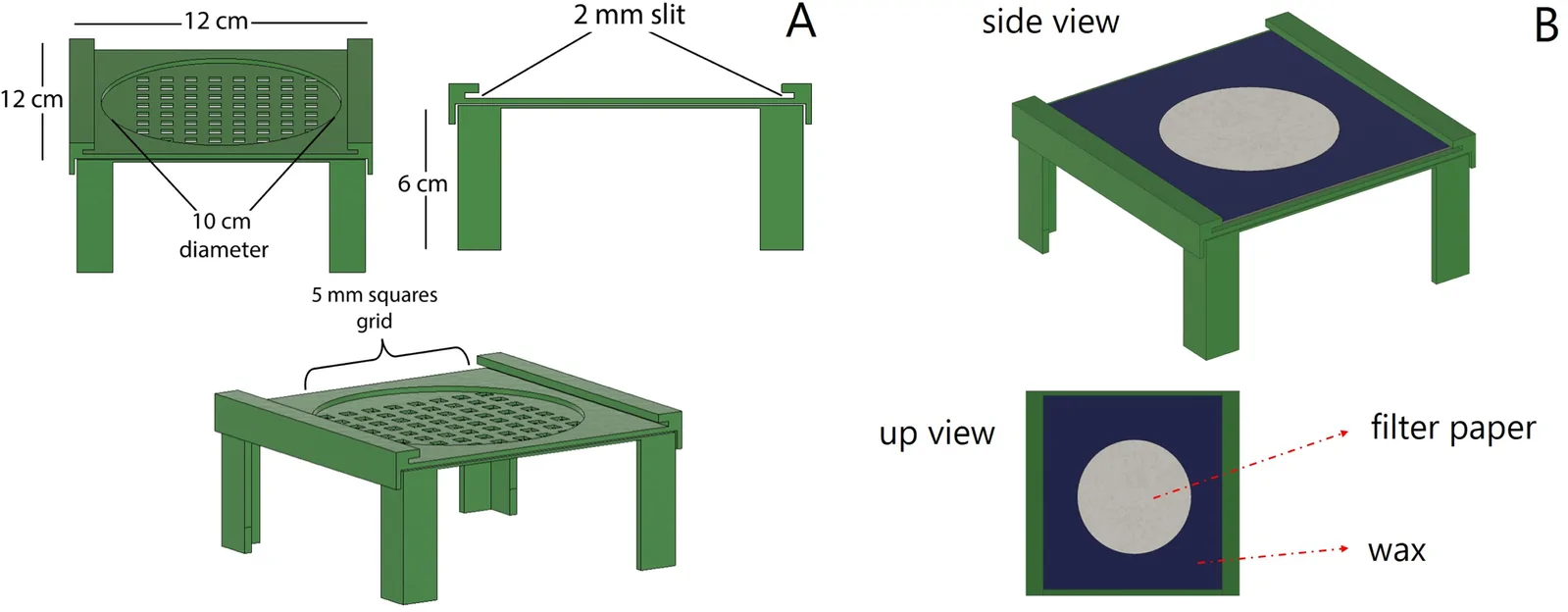
3D-printed accessory for paper-based platform support. (A) Dimensions and (B) assembly with the wax-printed paper-based platform.

The utilization of the paper-based platform follows a streamlined, customizable workflow that balances operational simplicity with scalable throughput. First, the platform size is selected and tailored according to the specific scale of the intended synthesis, ranging from small discs for microscale screening to large-scale 10 cm discs for gram-scale reactions. The paper substrate then undergoes a precise heat treatment, which melts the hydrophobic material, forcing it to penetrate the porous cellulose network and establish well-defined hydrophobic fluidic barriers. Following barrier formation, the platform is assembled onto its corresponding mechanical support to prevent sagging or tearing during liquid loading; smaller discs are taped, while larger scale-up platforms are locked securely into the custom 3D-printed grid frame. The reaction mixture is then loaded onto the designated reactive zone according to the specific chemical procedures outlined in the SI. Finally, the platform is left to dry completely at room temperature. Upon reaction completion and drying, the synthesized product naturally cracks and detaches from the paper matrix, enabling effortless harvesting and direct mass measurement into a collection vial without the need for traditional, solvent-heavy purification steps (Fig. 4).

**Fig. 4:**
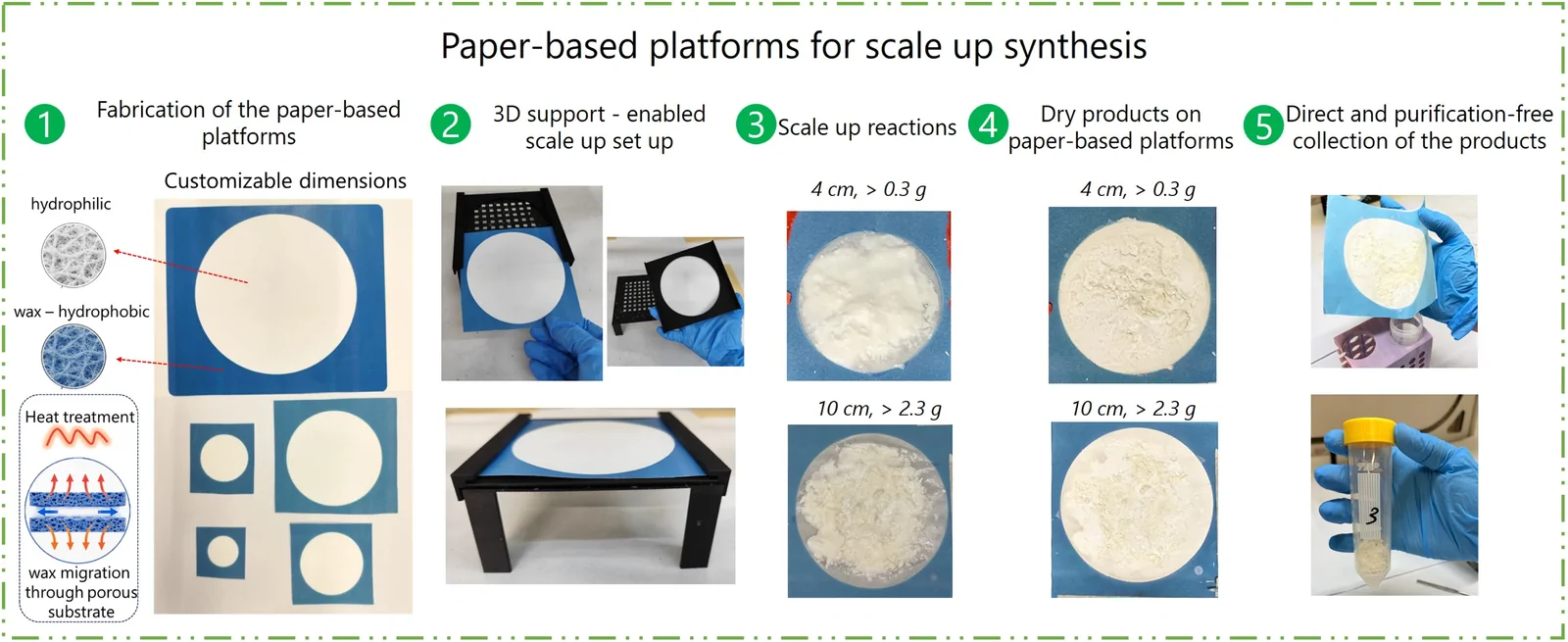
Workflow and experimental setup of the paper-based reaction platform. (1) Preparation of the paper-based platforms: design of the desired dimensions, printing, and heat treatment. (2) Integration of the 3D-printed support to enable reaction scale-up. (3) Performance and execution of the scale-up reaction. (4) Ambient drying of the synthesized products directly on the paper-based platforms. (5) Direct, seamless isolation of the final product into a container without requiring traditional purification steps.

### Comparative synthetic yield and scalability

To evaluate the preparative utility of the platform, the synthesis of squaramides was performed using four strategically selected diameters (0.5, 1, 4, and 10 cm). These scales were chosen as indicative examples to demonstrate the platform's versatility, with each step representing a nearly ten-fold increase in reaction volume (see Table S1). As an initial case study, we focused our attention on the synthesis of symmetric squaramides with benzylamine, a particularly simple and rapid transformation, to establish the operating limits of the platform. In our previous work, the model reaction was performed on 2 cm WF1 paper discs, using 20 mg of dimethyl squarate 1 (0.14 mmol) and 2 equiv. of benzylamine 2 (0.28 mmol) at a concentration of 0.5 M in EtOH/H_2_O mixture 1 : 1 corresponding to a total solvent volume of 280 µL. While these conditions were selected to demonstrate the efficiency of the transformation, in the present work a more detailed investigation of the solvent and porosity characteristic of the paper was undertaken. Based on the Table S1, 2 cm paper disc can accommodate up to approximately 300 µL of solvent, indicating that the previously employed conditions could be further adjusted to maximize the loading capacity of the support. Therefore, to maximize the use of the paper and provide hints for process intensification, we investigated whether the reaction concentration could be increased without compromising efficiency (Table 1).

**Table 1:** Synthesis of symmetric squaramides on different scale

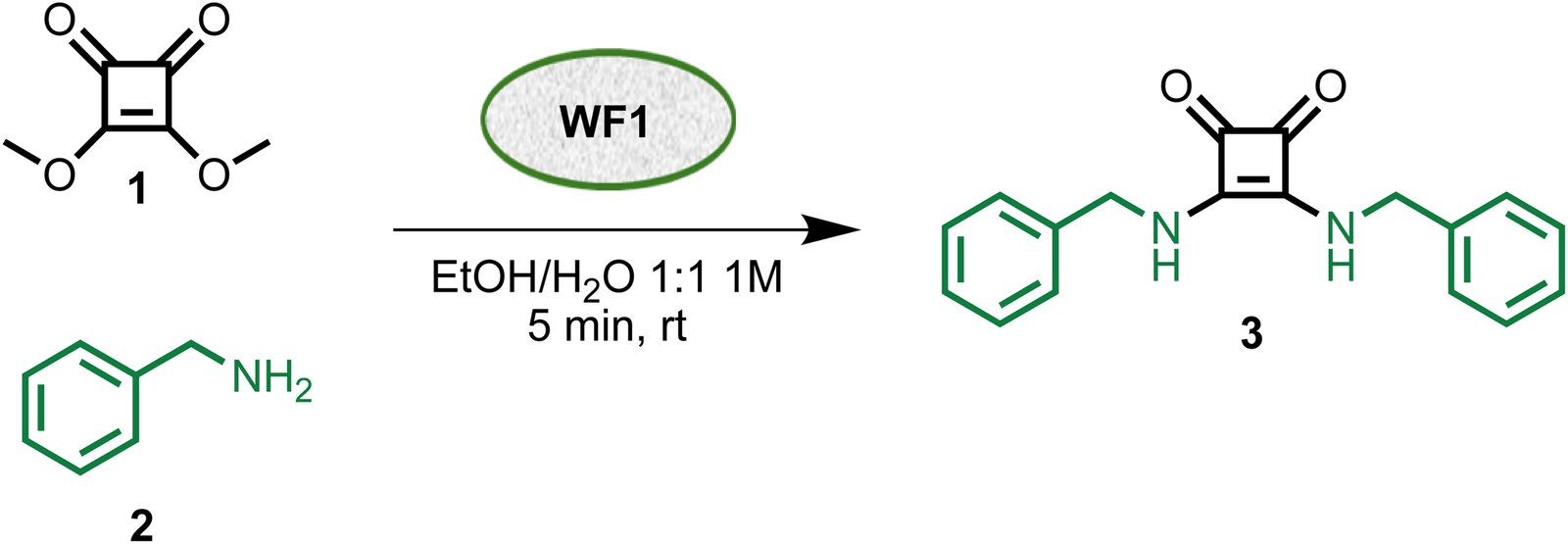				
Entry	Diameter of the filter paper (cm)	Yield 3 (%)	Isolated product (mmol)	Isolated mass (mg)
1	0.5	98	0.013	3.9
2	1	96	0.12	35
3	4	95	1.14	332
4	10	85	7.63	2230

Given the rapid kinetics of this transformation, the reaction was successfully optimized at 1 M, allowing a substantial reduction in solvent consumption, while maintaining excellent performance. The optimized conditions were subsequently applied across the selected paper sizes. Using 0.5 cm paper discs, 0.014 mmol of substrate was reacted in 14 µL of solvent, affording 3.9 mg of product in 98% yield (Entry 1). Scaling to 1 cm paper discs, 0.127 mmol of substrate was reacted in 127 µL of solvent, yielding 35 mg of product in 96% yield (Entry 2). In these small-scale experiments, solvent loadings slightly exceeded the values predicted by the scaling table. This deliberate choice was enabled by the rapid formation of the solid product, which limits lateral diffusion within the paper matrix and ensures efficient confinement of the reaction mixture despite increased solvent volumes. Further scale-up to 4 cm and 10 cm paper discs was performed using solvent and reagent amounts directly proportional to the scaling table (1.2 mL and 9 mL of solvent, corresponding to 1.2 and 9 mmol of substrate, respectively), providing 332 mg of product in 95% yield (Entry 3) and 2.23 g of product in 95% NMR yield with an 85% isolated yield after recovery (Entry 4). Remarkably, high reaction efficiency was maintained throughout the entire scale-up process, from milligram to gram scale, demonstrating the robustness of the platform. Furthermore, product isolation remained straightforward even at the largest scale, highlighting the practicality of this approach for preparative applications (Table 2).

**Table 2:** Synthesis of asymmetric squaramides (benzylamine-aniline) on different scale

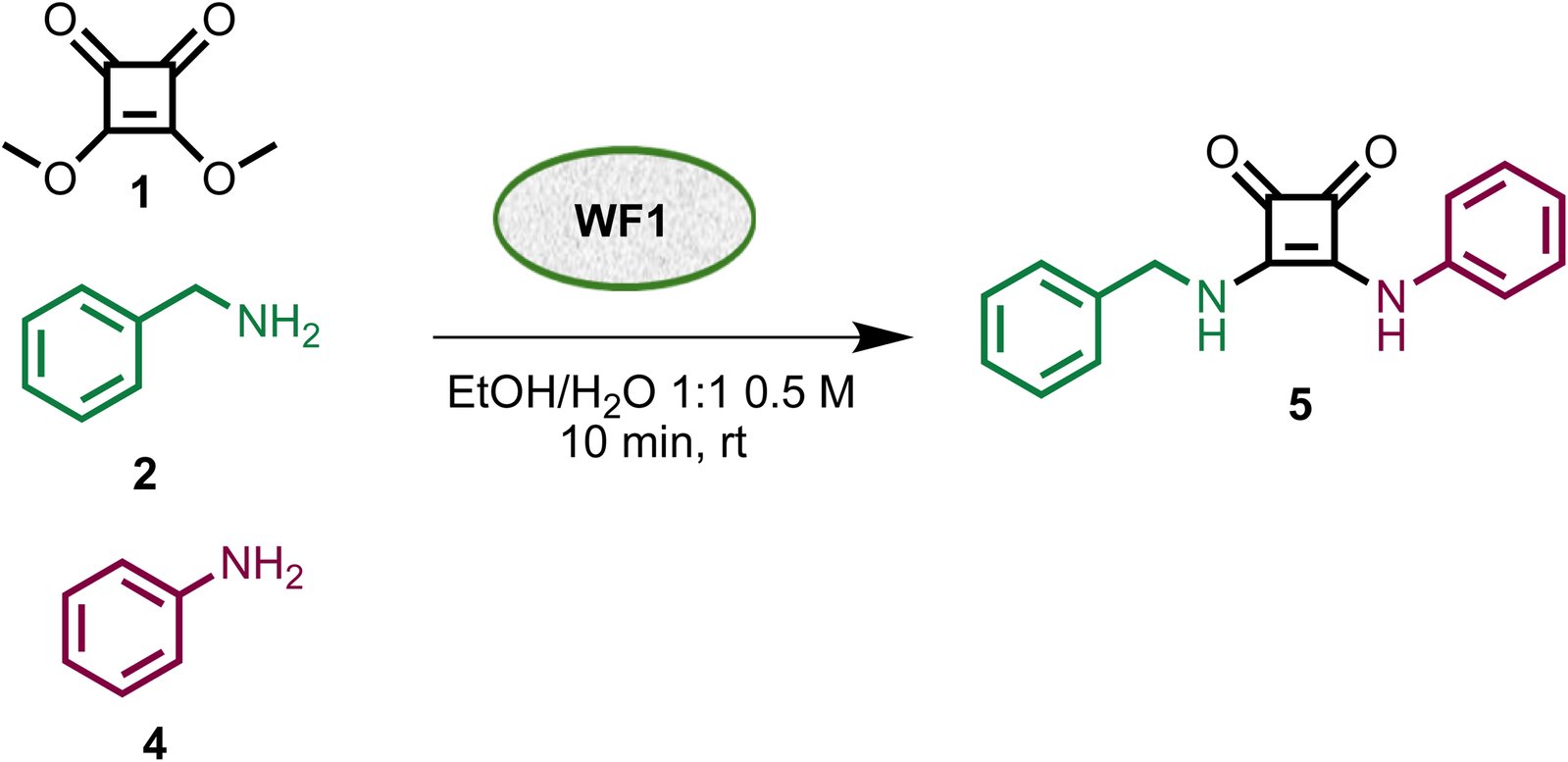				
Entry	Diameter of the filter paper (cm)	Yield 5 (%)	Isolated product (mmol)	Isolated mass (mg)
1	0.5	92	0.005	1.28
2	1	96	0.05	13
3	4	96	0.60	166

Having established the optimal set-up, we extended the same strategy to the synthesis of an asymmetric squaramide dimer. As we previously demonstrated in our work, the synthesis of asymmetric dimer from dimethyl squarate 1, benzylamine 2 and aniline 4, proceeds *via* a two-step sequence, involving initial formation of the corresponding mono-substituted intermediate followed by the benzylamine addition to afford the dimer 5. Building on the optimized conditions just identified for the symmetric dimer at 1 M concentration, we first attempted to apply the same conditions to the synthesis of the monomeric intermediate. However, incomplete conversion was observed, likely due to less efficient mixing within the paper matrix and the intrinsically slower kinetics of this transformation compared to the symmetric system. For this reason, we retained the previously established conditions reported in our earlier study. Under these conditions, the reaction proceeded efficiently across different paper sizes, further demonstrating the robustness and programmability of the platform. Using a 0.5 cm paper disc and 10 µL of solvent, the reaction was performed on a 0.005 mmol scale, affording the desired product in 92% yield, furnishing 1.28 mg (Entry 1). Using a 1 cm paper disc and 100 µL of solvent, as determined in our paper wettability study, the desired product was obtained in 96% yield, corresponding to 13 mg of isolated product (Entry 2). Comparable results were observed when using 4 cm paper discs, affording 166 mg of product in similar yield (Entry 3). The slightly lower yield in 0.5 cm paper compared to the larger disc is likely due to less efficient mixing on the smaller reaction surface. To improve mixing, an additional 10 µL of solvent was added 5 min after the first step, before the addition of benzylamine. Importantly, the sequential addition process yielded exclusively the targeted unsymmetrical squaramide, with no detectable formation of symmetrical dimer side-products or unreacted intermediate by ^1^H NMR analysis. The reaction was not performed on 10 cm paper discs to avoid unnecessary consumption of starting material, as the successful scale-up behavior had already been validated across smaller paper platform sizes in agreement with the scaling parameters (Table 3).

**Table 3:** Synthesis of asymmetric squaramides (aliphatic-aniline) on different scale

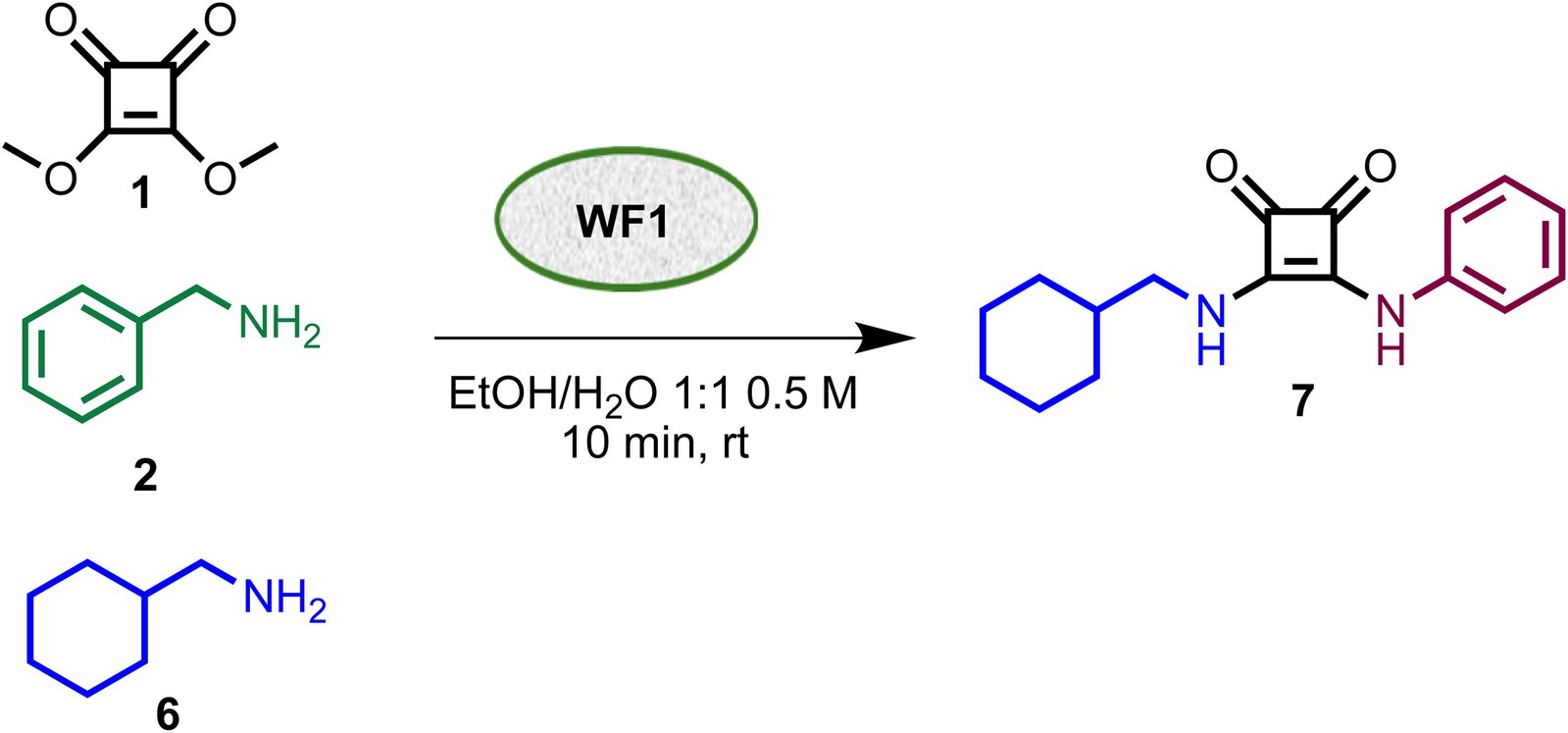				
Entry	Diameter of the filter paper (cm)	Yield 7 (%)	Isolated product (mmol)	Isolated mass (mg)
1	0.5	84	0.004	1.19
2	1	89	0.044	12.6
3	4	80	0.46	132

Finally, we investigated the coupling of aliphatic amines with anilines using the protocol optimized for the asymmetric aniline–benzylamine dimer. Maintaining identical volumetric parameters across scale transitions, the reaction between dimethyl squarate, cyclohexylmethanamine and aniline demonstrated high efficiency across all three paper dimensions. Particularly, 0.5 cm yielded 1.19 mg (0.004 mmol, 84% yield), which scaled smoothly to 12.6 mg (0.044 mmol, 89% yield) in the 1.0 cm diameter, and ultimately afforded 132 mg (0.46 mmol, 80% yield) in the 4.0 cm filter paper.

The scalability of the symmetric dimer paper-based scale up was evaluated by increasing the platform diameter from 0.5 to 10 cm, corresponding to an increase in isolated product mass from 3.9 mg to 2.23 g. This represents an approximately 570-fold increase in product mass, corresponding to nearly three orders of magnitude in reaction scale. Despite this substantial scale-up, the isolated reaction yield remained consistently high, decreasing only modestly from 98% at the smallest scale to 85% at the largest scale (Fig. 5B). Accordingly for the asymmetric dimer 5 scale up synthesis we obtained similar results, with a yield ranging between 92–96% across the different platforms sizes and products mass (Fig. 5C). For the asymmetric dimer 7 a slightly lower yield ranging from 80–89 was obtained, across the scale-up platforms (Fig. 5D).

**Fig. 5:**
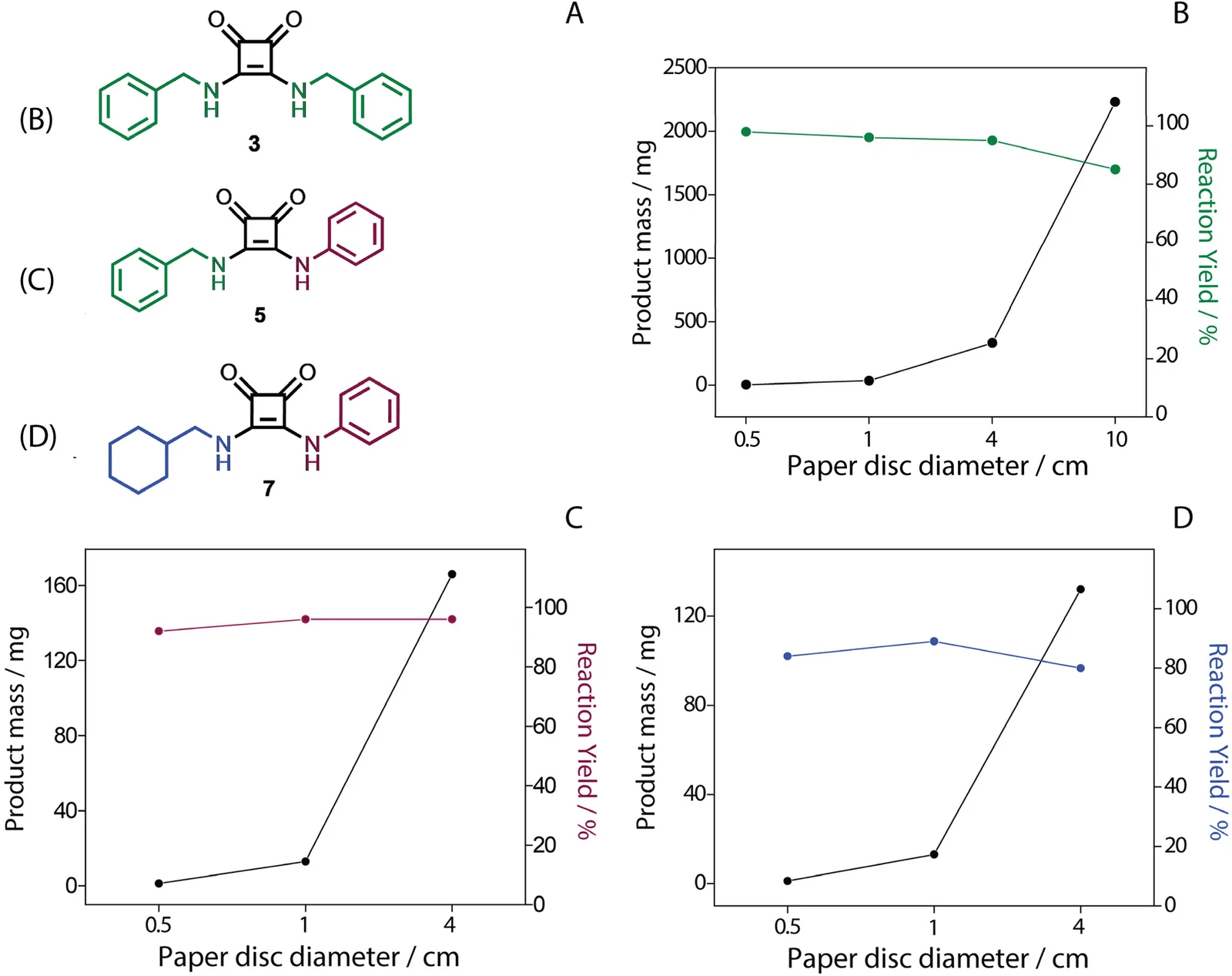
(A) Chemical structures of the symmetrical (3) and assymmetric products (5, 7) corresponding to the obtained graphs. (B) Effect of paper platform diameter on reaction scale and yield for the symmetrical products (3). Product mass (left *y*-axis) and isolated reaction yield (right *y*-axis) obtained using paper platforms with diameters ranging from 0.5 to 10 cm. (C) Effect of paper platform diameter on reaction scale and yield for the asymmetric product 5. Product mass (left *y*-axis) and isolated reaction yield (right *y*-axis) obtained using paper platforms with diameters ranging from 0.5 to 4 cm. (D) Effect of paper platform diameter on reaction scale and yield for the asymmetric product 7. Product mass (left *y*-axis) and isolated reaction yield (right *y*-axis) obtained using paper platforms with diameters ranging from 0.5 to 4 cm.

The ability to maintain high product yields over such a broad range of reaction scales demonstrates the robustness and scalability of the paper-based reaction platform. The slight decrease in yield at the largest scale, for the symmetric dimer, is expected given the increased reaction volume and diffusion distances but remains remarkably low considering the nearly three-order-of-magnitude increase in product output. These results highlight the excellent scalability of the approach and confirm that the paper platform provides a homogeneous reaction environment capable of supporting efficient synthesis from milligram to gram-scale production. This behavior is particularly advantageous for the synthesis of symmetric compounds, where maintaining high conversion and selectivity during scale-up is essential for achieving high isolated yields without requiring modifications to the reaction protocol.

### Green metrics

Finally, green metrics were assessed for the symmetric and asymmetric dimers to evaluate the environmental impact, performance, and sustainability of the paper-based protocol. The parameters contributing to the green metrics of the synthetic route were determined, as illustrated in Fig. 6 and in Fig. 7 for the model compound dimer 3 and dimer 5 (see SI, Fig. S5 for Green Metrics of asymmetric dimer 7). We selected a 4 cm diameter as the model system, as it lies between the small and very large scales investigated. All metrics were in agreement with our previous work. Atom economy and atom efficiency yielded positive results. Carbon efficiency and reaction mass efficiency were close to 100% across all processes. A crucial parameter within the environmental assessment is the EcoScale score. Specifically, regarding the symmetrical dimer, the EcoScale score reached 88.5 (>75, excellent; >50, acceptable; <50, inadequate), in accordance with our previous work. This outcome highlights the outstanding productivity and reproducibility of the designed methodology.

**Fig. 6:**
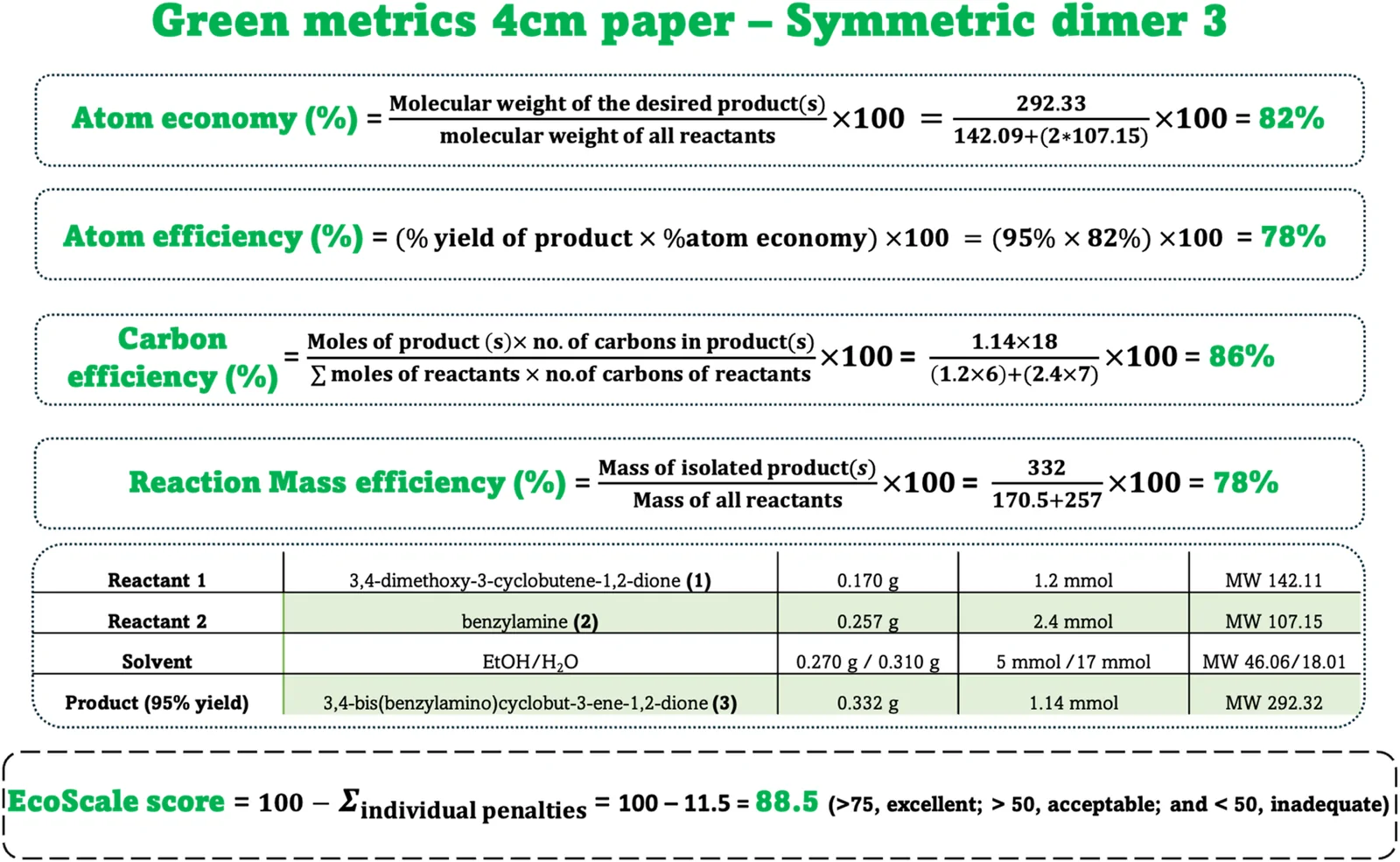
Green metrics components detailed calculation for the model compound 3.

**Fig. 7:**
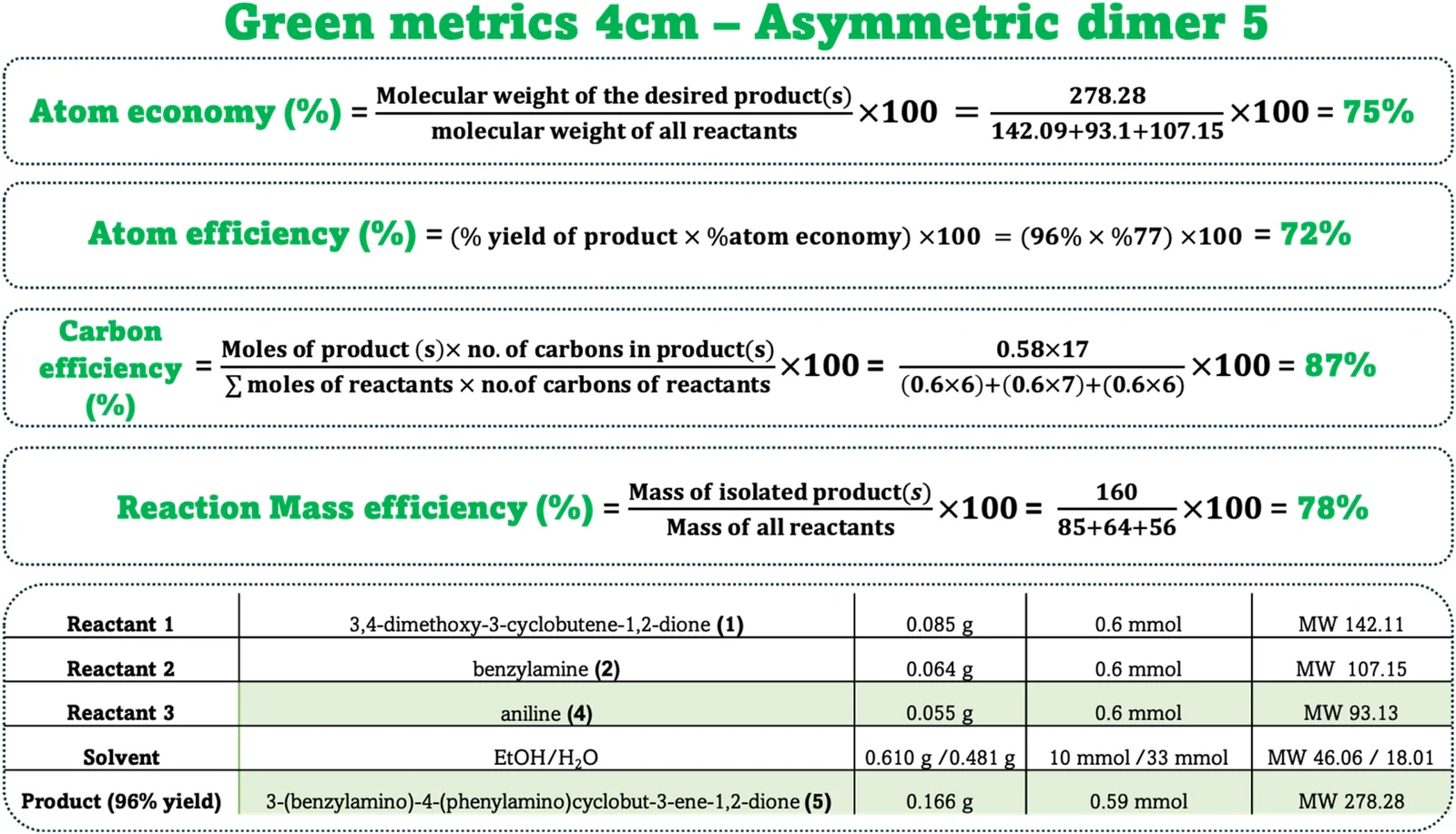
Green metrics components detailed calculation for the model compound 5.

Focusing tailored attention on the E-factor, one of the most important metrics in Green Chemistry, it is worth highlighting that this parameter remains remarkably stable upon scale-up. This constancy stands as one of the most prominent and impactful outcomes of the present work. This is directly attributable to the use of an eco-friendly solvent combined with the paper-based reaction platform, which promotes a rapid process at room temperature while completely bypassing tedious isolation and purification steps.

Focusing tailored attention on the E-factor, one of the most important metrics in Green Chemistry, it is worth highlighting that this parameter remains remarkably low and/or stable upon scale-up. More specifically, E-factor metric was calculated for both symmetric (Fig. S2) and asymmetric dimers (Fig. S3) across all the paper-based platform dimension ranges that were used for the scale up procedure (Fig. 8). For the symmetric dimer E-factor remains both low and constant in all scale up reaction, while for the asymmetric ones this is observed in the higher scale up reactions. This green metric constancy stands as one of the most prominent and impactful outcomes of the present work. This is directly attributable to the use of an eco-friendly solvent combined with the paper-based reaction platform, which promotes a rapid process at room temperature while completely bypassing tedious isolation and purification steps.

**Fig. 8:**
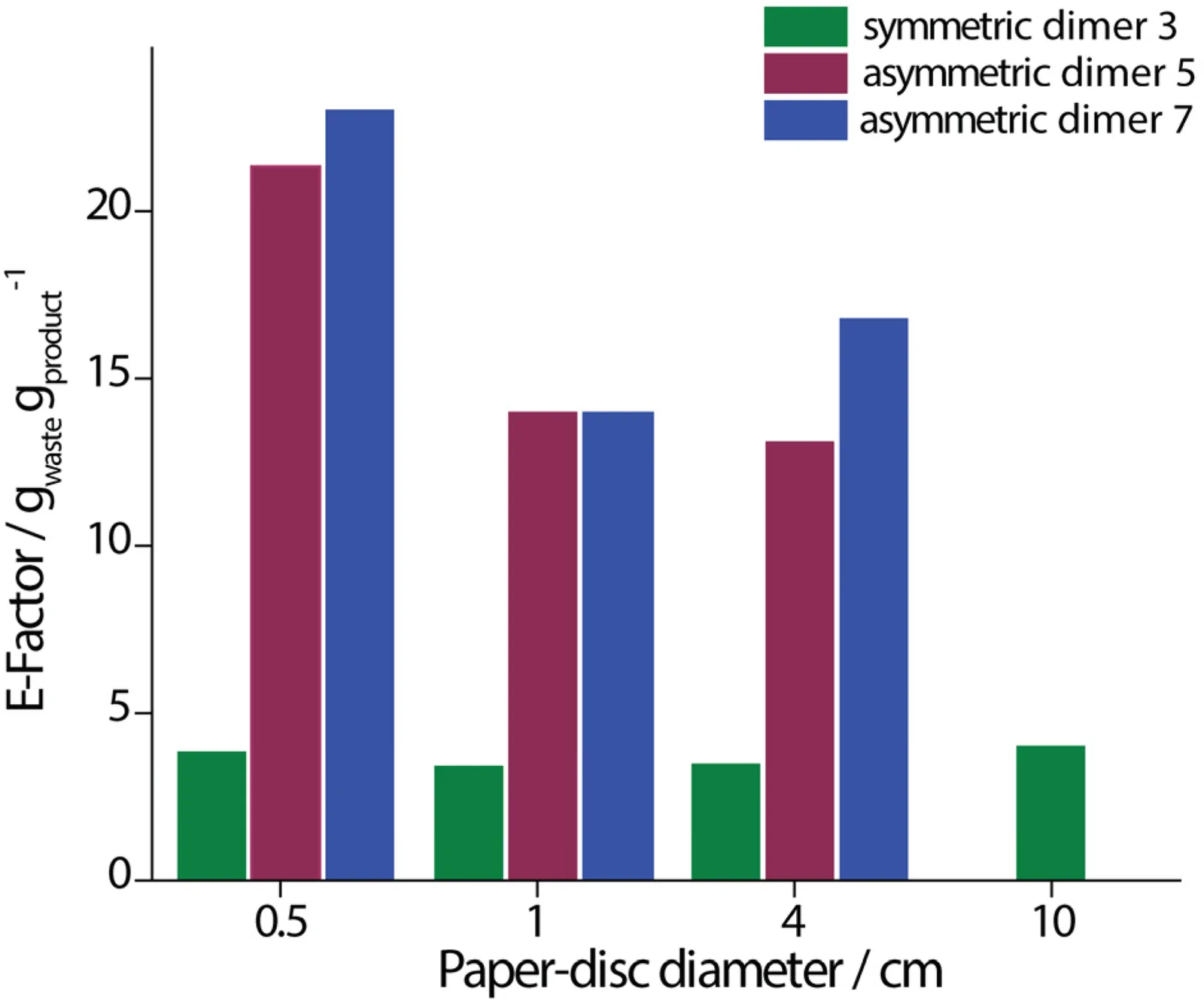
E-factor assessment for both symmetric (3) and asymmetric dimers (5, 7) across all scale up range.

### Limitations of the paper-based scale-up platform

Despite the promising scalability demonstrated in this study, several limitations should be taken into account for future refinement and improvement of the methodology. First, the solvent compatibility of cellulose-based substrates remains a critical factor. While the platform performs effectively with aqueous and aqueous-ethanolic systems, prolonged exposure to certain organic solvents (*e.g.*, DMF, DMSO, THF, dichloromethane, chloroform, acetone, and toluene) may compromise paper integrity, alter fluid transport properties, or weaken wax barriers. Consequently, solvent selection remains more restricted than in conventional glass reactors. Future work will aim at investigating alternative barrier fabrication strategies with enhanced chemical resistance, including chalk wax and silicon-based patterning, polymeric coatings, additive-manufacturing approaches, direct deposition of hydrophobic materials,^12,26–28^ and solvent-resistant barrier architectures that may provide greater mechanical robustness and broader solvent compatibility than conventional wax printing.

Second, although scale-up was achieved up to ∼10 mL reaction volume and multi-gram product output, translation to kilogram-scale manufacturing will likely require numbering-up strategies involving multiple parallel paper reactors rather than further enlargement of individual discs. The development of modular stacked reactors, continuous-flow paper assemblies, and automated reagent delivery systems could facilitate industrially relevant throughput while preserving the simplicity and sustainability of the platform.

Third, evaporation losses may become increasingly significant for highly volatile solvents despite the use of the 3D-printed support system. More sophisticated reactor housings fabricated through additive manufacturing could provide improved solvent retention and environmental control. Additionally, reactions requiring vigorous mixing, elevated pressures, high temperatures, or heterogeneous slurries may be challenging to implement using cellulose alone. Future studies should therefore evaluate alternative porous substrates,^29–31^ including wool, textile fibers, and hybrid cellulose-polymer composites, to expand the operational window of paper-based synthesis while retaining capillary-driven transport and low-cost fabrication.

## Conclusion

In summary, this work redefines the conventional view of paper microreactors as mere single-use screening tools by introducing a revolutionary, geometry-driven scale-up paradigm. Through a precise, programmable framework, we seamlessly bridged the gap between microscale optimization and multi-gram preparative manufacturing on a single, low-cost platform. The profound novelty of this approach lies in establishing a quantitative, predictable bridge between cellulosic physical properties and macro-scale chemical output, transforming paper architecture into a highly programmable synthetic platform where simple geometric expansion directly governs fluidic capacity and mass yield. The transformative nature of our protocol is further boosted by the substantially unaltered green metrics registered in the transition from small scale application.

Combining biodegradable Whatman filter paper with a stabilizing, 3D-printed modular scaffold yielded a highly reproducible system with excellent green metrics. This innovative setup successfully overcomes the structural fragility typically associated with paper substrates, ensuring robust mechanical integrity even during solid product formation and precipitate loading. By utilizing passive capillary action, this eco-friendly methodology maximizes atom economy, dramatically reduces solvent waste, streamlines crude purification steps, and completely eliminates the need for expensive, energy-intensive external pumping infrastructure.

Ultimately, this novel, highly cost-effective technology positions cellulose not merely as a simple analytical substrate, but as a robust, macro-scale synthetic reactor capable of competing with traditional glassware and even a microscale screening platform. By proving that high-value, solid-yielding reactions like squaramide synthesis can be scaled predictably spanning three orders of magnitude, this work offers an accessible, transformative, and highly practical frontier for decentralized, localized, and low-resource chemical manufacturing, effectively aligning preparative organic synthesis with the core mandates of green chemistry. Future studies will investigate alternative wax materials and paper substrates to expand solvent compatibility beyond the aqueous conditions examined here. Further validation with a broader range of reactions will also be needed to establish the versatility of the platform.

## Conflicts of interest

There are no conflicts to declare.

## Supplementary Material

RA-016-D6RA09121K-s001

## Data Availability

The data supporting the findings of this study are included in the paper and the supplementary information (SI), and are also available from the corresponding authors upon reasonable request. Supplementary information: full experimental procedures, characterization data of compounds, guided tables and copies of ^1^H and ^13^C NMR spectra. See DOI: https://doi.org/10.1039/d6ra09121k.
