# Supplementary material for: Programmable and sustainable scaling up of squaramide-based compounds through customizable paper-based platforms
Source: RSC Adv. 2026 Oct 20. Online ahead of print. doi: 10.1039/d6ra09121k (PMC13681196; doi:10.1039/d6ra09121k)
Supplement: Supplementary file 1 [file RA-OLF-D6RA09121K-s001.pdf]

This journal is © The Royal Society of Chemistry

Programmable and sustainable scaling up of squaramide-based compounds through customizable paper-based platforms

Panagiota M. Kalligosfyri[a, $,*] Antonella Ilenia Alfano[a, $,*], Stefano Cinti[a,b,c,d,#], Margherita Brindisi[a, #]

[a] Department of Pharmacy (DoE 2023-2027), University of Naples Federico II, via D. Montesano 49, 80131, Naples, Italy;

[b] Sbarro Institute for Cancer Research and Molecular Medicine, Center for Biotechnology, College of Science and Technology, Temple University, Philadelphia, Pennsylvania 19122, United States;

[c] Bioelectronics Task Force at University of Naples Federico II, Naples, 80126, Italy;

[d] Department of Chemistry, Faculty of Science, Chulalongkorn University, Bangkok 10330, Thailand.

[$] Co-first authors.
[*] Corresponding authors
[#]Co-last authors

Table of contents

General materials and methods	S1
General experimental protocols	S2
Experimental correlation of paper disc diameter, wettability, and liquid loading volume	S3
Microscopic characterisation of the paper-based reaction platform	S4
Characterization data	S5
Green metrics calculation	S7
Copies of NMR Spectra	S9
References	S12

References	S10

General materials and methods

Unless otherwise stated, all solvents were purchased from Sigma-Aldrich and used without further purification. Also, unless otherwise stated, all substrates and reagents were purchased from Zentek or Sigma-Aldrich and used as received. Red food dye (20x diluted), obtained from local convenient store, was used for the experimental investigation of the wettability and the loading volume across all paper discs dimensions (0.5-10 cm).

Preparation of the paper-based platforms

Whatman filter paper Grade 1 (Sigma-Aldrich, St.Louis, MO, USA) was employed for the fabrication of the paper-based reaction platforms. The platforms were designed by defining the desired disc diameter using Adobe Illustrator (version 2019). The patterns were then wax-printed using a ColorQube 8880 solid-ink printer (Xerox, USA). This printing step was carried out to define hydrophobic barriers within the paper substrate and to enable controlled liquid handling and confinement of reaction zones. Following printing, the patterned paper discs were pretreated in an oven at 100 °C for 1 min to ensure complete melting and permeation of the wax through the full thickness of the filter paper, thereby forming well-defined hydrophobic barriers on both sides of the substrate.

Preparation of the 3D-printed paper-based platform's support

For structural support and improved mechanical stability during reaction setup, a three-dimensional (3D) printed holder was designed using Autodesk Fusion 360 software. The support platform was fabricated using a Bambu Lab X1 Carbon 3D printer (Bambu Lab, Shanghai, China) with basic black PLA filament (1.75 mm; Bambu Lab, Shenzen, China), with the exact dimensions as shown in Figure 1.

Reaction monitoring and product characterization

^{1}H-NMR spectra were recorded on 400 instrument and are reported relative to the residual solvent: DMSO-d_6 (δ2.50 ppm). A weighed amount of analyte (about 5.0–10.0 mg) was dissolved in 600 µL of deuterated dimethyl sulfoxide (DMSO-d_6). The mixture was transferred into a 5 mm NMR tube and the spectra were acquired on a Bruker Advance 400 MHz and 700 MHz spectrometer by using the residual signal of the deuterated solvent as internal standard. Splitting patterns are described as singlet (s), doublet (d), triplet (t), quartet (q) and broad (br); the values of chemical shifts (δ) are given in ppm and coupling constants (*J*) in Hertz (Hz). NMR data were processed with MestreNova (Version 8.1.1, Mestrelab Research).

General experimental protocols

General procedure A for compound **3**:

A solution of 3,4-dimethoxy-3-cyclobutene-1,2-dione in a solution of EtOH/H_2O 1:1 was prepared (1 M). Once total solubility was achieved, the solution was deposited on the appropriate filter disc. Then, the benzylamine (2 equiv.) was added dropwise onto the filter disc. After 5 min at room temperature, the reaction was completed. The 3D-printed housing was used in the 10 cm-scale procedure to ensure the stability of the paper-based synthesis platform. The completely dried product was removed from the filter disc without further purification step and directly analysed with NMR. Each compound was weighed on the analytical balance to obtain the final yield.

General procedure B for compounds **5** and **7**:

A solution of 3,4-dimethoxy-3-cyclobutene-1,2-dione in a solution of EtOH/H_2O 1:1 was prepared (0.6 M). Once total solubility was achieved, the solution was deposited on the appropriate filter disc. Then, the aniline (1 equiv.) was dissolved in EtOH/H2O 1:1 in order to achieve 0.5 M concentration during the reaction and added dropwise onto the filter disc. After 5 min at room temperature under the fume hood, subsequent step involved adding a mixture solution of EtOH/H_2O 1:1 (0.6 M) to solubilize the initial product, followed by the dropwise addition of the benzylamine (1 equiv.) or cyclohexanemethylamine in EtOH/H_2O 1 in order to achieve 0.5 M concentration during the reaction. After 5 min at room temperature under the fume hood, the reaction was completed. The final dried product was removed from the filter disc without further purification step and directly analysed with. All the paper containing the compounds were left under the fume hood overnight. By the following day, the solvent had completely evaporated or been absorbed, and the compounds were ready to be transferred into vials, without efforts. Each compound was then weighed on the analytical balance to obtain the final yield.

Experimental correlation of paper disc diameter, wettability, and liquid loading volume

Table S1 summarizes the experimental correlation between paper-disc diameter, wettability, and the corresponding liquid loading volume used in this study. As the paper-disc diameter increased, both the wettability and the maximum loading volume increased, reflecting the higher liquid-holding capacity of larger paper platforms. These values served as a practical guide for selecting appropriate sample volumes based on the dimensions of the paper discs.

Table S1. Guide for the correlation of the paper platform dimensions with the experimental load volume.

Paper-disc diameter (cm)	Wettability (uL)	Loaded volume (mL)
**0.5**	2.4	0.010
**0.6**	3	0.018
**0.7**	4.5	0.037
**0.8**	6	0.060
**0.9**	7.5	0.070
**1.0**	18	0.100
**2.0**	50	0.300
**3.0**	60	0.520
**4.0**	160	1.200
**5.0**	250	2.300
**6.0**	300	2.800
**7.0**	490	4.000
**8.0**	1000	5.000
**9.0**	1500	7.000
**10.0**	1800	9.000

Microscopic characterisation of the paper-based reaction platform

To assess the interaction between the paper-based platform and the reaction components, microscopic analysis was performed using an Exacta Optech microscope equipped with a Euromex Scientific Camera (Exacta Labcenter SpA, Italy). Images captured before and after the reaction tracked the platform's state. Upon drying, the product naturally cracked and detached from the paper, allowing effortless transfer into a vial without mechanical force. Post-removal analysis revealed no structural damage, swelling, or morphological alterations. Consistent with our previous work[1] these results confirm the paper substrate maintains excellent structural integrity and stability throughout the process, despite minor residual surface particles.

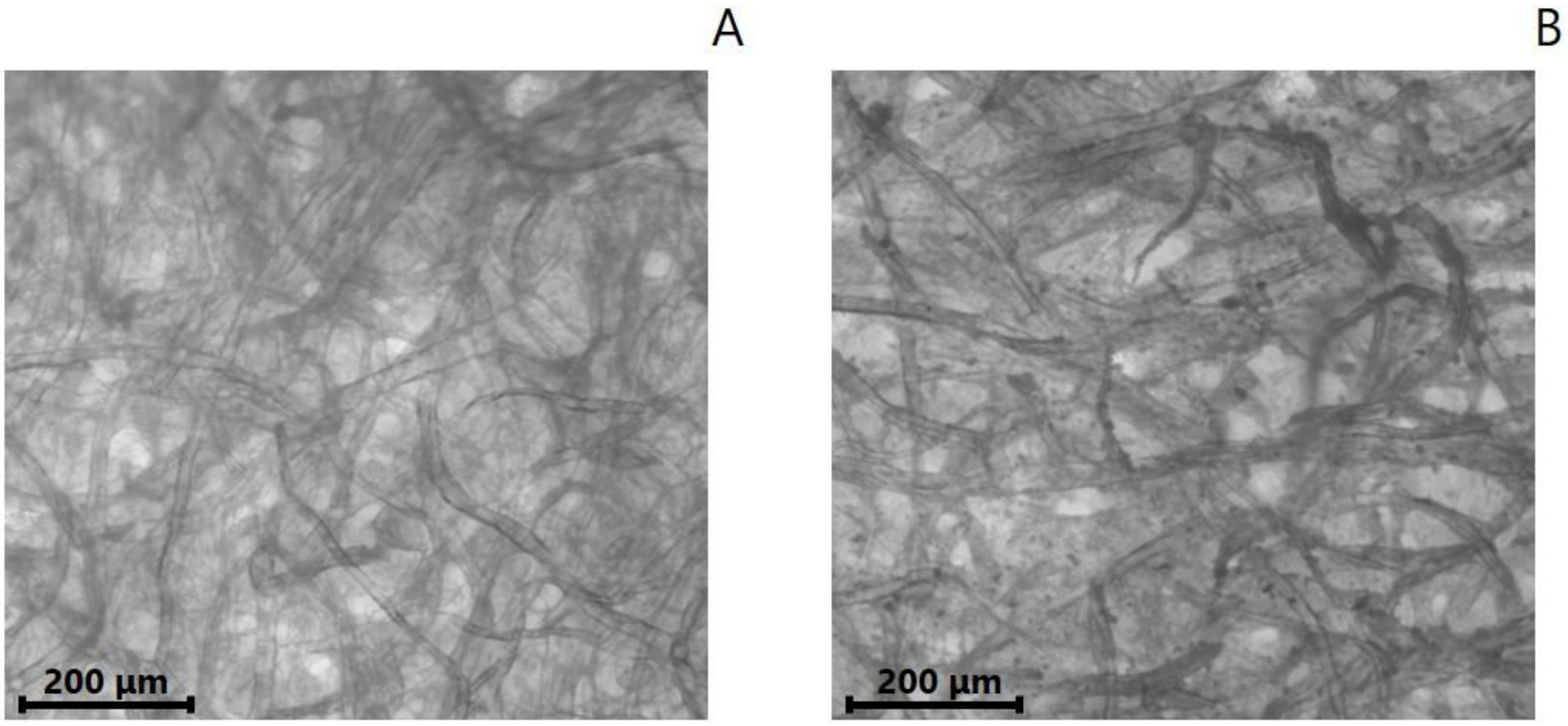

Figure S1: Microscope images of the paper-based platform A) before and B) after the reaction and product removal.

Characterization data

3: 3,4-bis(benzylamino)cyclobut-3-ene-1,2-dione

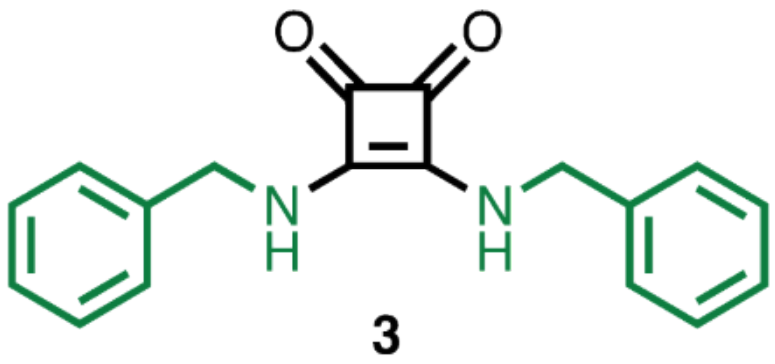

3

Following the general procedure A, using different amounts of 3,4-dimethoxy-3-cyclobutene-1,2-dione and benzylamine (2 equiv.), yields between 98% and 85% of **3** were obtained as white solid. The spectroscopic data are in line with those previously reported in literature.[1]

^{1}H NMR (400 MHz, DMSO) δ 7.73 (s, 2H), 7.37 (dd, *J* = 15.9, 5.8 Hz, 5H), 7.32 – 7.28 (m, 5H), 4.71 (d, *J* = 6.8 Hz, 4H).

^{13}C NMR (101 MHz, DMSO) δ 182.64, 167.53, 138.91, 128.64, 127.49, 127.41, 46.80.

MS (ES+) calc for $C_{18}H_{16}N_2O_2$ 293.1 $(M+H)^+$, found 293.1 $(M+H)^+$.

5: 3-(benzylamino)-4-(phenylamino)cyclobut-3-ene-1,2-dione

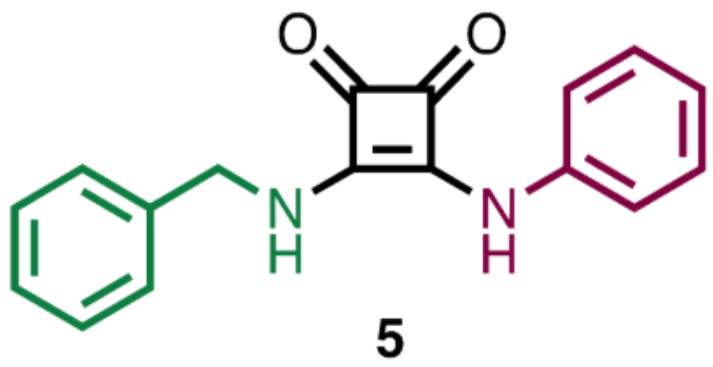

5

Following the general procedure B, using different amounts of 3,4-dimethoxy-3-cyclobutene-1,2-dione, aniline (1 equiv.) and benzylamine (1 equiv.), yields between 92% and 96% of **5** were obtained as white solid. The spectroscopic data are in line with those previously reported in literature.[1]

^{1}H NMR (400 MHz, DMSO-d$_6$) δ 9.67 (s, 1H), 8.04 (s, 1H), 7.44 – 7.39 (m, 6H), 7.35 (t, *J* = 7.9 Hz, 3H), 7.02 (t, *J* = 7.3 Hz, 1H), 4.81 (d, *J* = 6.0 Hz, 2H).

^{13}C NMR (101 MHz, DMSO) δ 184.07, 180.42, 168.89, 163.85, 138.99, 138.50, 129.42, 128.82, 127.74, 127.70, 122.78, 118.09, 47.27.

MS (ES+) calc for $C_{17}H_{14}N_2O_2$ 279.1 $(M+H)^+$, found 279.1 $(M+H)^+$.

7: 3-((cyclohexylmethyl)amino)-4-(phenylamino)cyclobut-3-ene-1,2-dione:

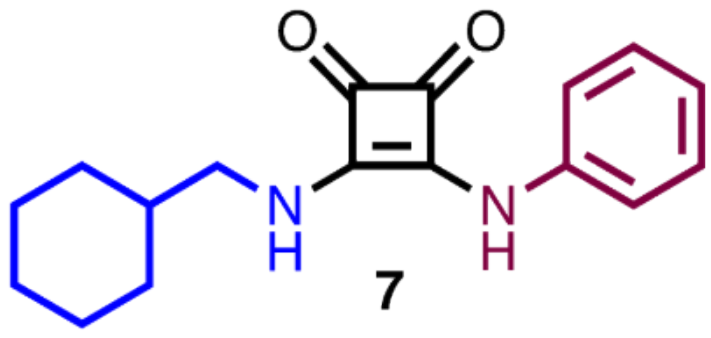

7

Following the general procedure B, using different amounts of 3,4-dimethoxy-3-cyclobutene-1,2-dione, aniline (1 equiv.) and cyclohexylmethanamine (1 equiv.), yields between 80% and 89% of **7** were obtained as white solid. The spectroscopic data are in line with those previously reported in literature.[1]

^{1}H NMR (400 MHz, DMSO-d$_6$) δ 9.63 (s, 1H), 7.70 (s, 1H), 7.45 (d, *J* = 8.0 Hz, 2H), 7.33 (t, 2H), 7.02 (t, *J* = 7.3 Hz, 1H), 3.47 (t, *J* = 6.6 Hz, 2H), 1.71 (d, *J* = 8.3 Hz, 4H), 1.19 (dd, *J* = 20.9, 11.4 Hz, 4H), 1.01 – 0.91 (m, 3H).

^{13}C NMR (101 MHz, DMSO) δ 184.45, 180.58, 169.91, 163.84, 139.56, 129.82, 123.00, 118.38, 50.14, 39.19, 30.11, 26.36, 25.71.

Green metrics calculation

The calculation of Green Metrics for compound 7 and E-factors across different reaction scale for the symmetric and asymmetric dimers **3**, **5** and **7** respectively, are shown in Figure S2, S3, S4 and S5.

E-factor – Symmetric dimer 3

$$\text{E-factor 0.5cm} = \frac{\text{Total waste (g)}}{\text{Total product (g)}} = \frac{(0.0055+0.007+0.002+0.003)-0.0039}{0.0039} = 3.48\ g_{waste}/g_{product}$$

$$\text{E-factor 1 cm} = \frac{\text{Total waste (g)}}{\text{Total product (g)}} = \frac{(0.05+0.06+0.018+0.027)-0.035}{0.035} = 3.43\ g_{waste}/g_{product}$$

$$\text{E-factor 4 cm} = \frac{\text{Total waste (g)}}{\text{Total product (g)}} = \frac{(0.47+0.60+0.17+0.25)-0.332}{0.332} = 3.48\ g_{waste}/g_{product}$$

$$\text{E-factor 10 cm} = \frac{\text{Total waste (g)}}{\text{Total product (g)}} = \frac{(3.50+4.50+1.28+1.92)-2.23}{2.23} = 4.02\ g_{waste}/g_{product}$$

Figure S2: E-factor calculation for symmetric dimer **3**.

E-factor – Asymmetric dimer 5

$$\text{E-factor 0.5 cm} = \frac{\text{Total waste (g)}}{\text{Total product (g)}} = \frac{(0.004+0.005+0.0007+0.0005+0.0004+0.004+0.005+0.004+0.005)-0.00128}{0.00128} = 21.34\ g_{waste}/g_{product}$$

$$\text{E-factor 1 cm} = \frac{\text{Total waste (g)}}{\text{Total product (g)}} = \frac{(0.04+0.05+0.007+0.005+0.004+0.04+0.05)-0.013}{0.013} = 14.0\ g_{waste}/g_{product}$$

$$\text{E-factor 4 cm} = \frac{\text{Total waste (g)}}{\text{Total product (g)}} = \frac{(0.47+0.60+0.085+0.064+0.055+0.47+0.60)-0.166}{0.166} = 13.12\ g_{waste}/g_{product}$$

Figure S3: E-factor calculation for asymmetric dimer **5**.

E-factor – Asymmetric dimer 7

$$\text{E-factor 0.5 cm} = \frac{\text{Total waste (g)}}{\text{Total product (g)}} = \frac{(0.004+0.005+0.0007+0.0005+0.0004+0.004+0.005+0.004+0.005)-0.00119}{0.00119} = 23.03\ g_{waste}/g_{product}$$

$$\text{E-factor 1 cm} = \frac{\text{Total waste (g)}}{\text{Total product (g)}} = \frac{(0.04+0.05+0.007+0.005+0.004+0.04+0.05)-0.013}{0.013} = 14.0\ g_{waste}/g_{product}$$

$$\text{E-factor 4 cm} = \frac{\text{Total waste (g)}}{\text{Total product (g)}} = \frac{(0.61+0.48+0.085+0.068+0.055+0.61+0.48)-0.132}{0.132} = 16.8\ g_{waste}/g_{product}$$

Figure S4: E-factor calculation for asymmetric dimer **7**.

Green metrics 4cm – Asymmetric dimer 7

$$\textbf{Atom economy (\%)} = \frac{\text{Molecular weight of the desired product}(s)}{\text{molecular weight of all reactants}} \times 100 = \frac{284.35}{142.09+93.1+113.20} \times 100 = \textbf{82\%}$$

$$\textbf{Atom efficiency (\%)} = (\%\ \text{yield of product} \times \%\text{atom economy}) \times 100 = (96\% \times \%77) \times 100 = \textbf{66\%}$$

$$\textbf{Carbon efficiency (\%)} = \frac{\text{Moles of product }(s) \times \text{ no. of carbons in product}(s)}{\sum \text{moles of reactants} \times \text{no.of carbons of reactants}} \times 100 = \frac{0.46 \times 17}{(0.6\times6)+(0.6\times7)+(0.6\times6)} \times 100 = \textbf{68\%}$$

$$\textbf{Reaction Mass efficiency (\%)} = \frac{\text{Mass of isolated product}(s)}{\text{Mass of all reactants}} \times 100 = \frac{132}{85+68+56} \times 100 = \textbf{64\%}$$

**Reactant 1**	3,4-dimethoxy-3-cyclobutene-1,2-dione **(1)**	0.085 g	0.6 mmol	MW 142.11
**Reactant 2**	cyclohexylmethanamine **(6)**	0.068 g	0.6 mmol	MW 113.20
**Reactant 3**	aniline **(4)**	0.055 g	0.6 mmol	MW 93.13
**Solvent**	EtOH/H_2O	0.610 g /0.481 g	10 mmol /33 mmol	MW 46.06 / 18.01
**Product (80% yield)**	3-((cyclohexylmethyl)amino)-4-(phenylamino)cyclobut-3-ene-1,2-dione **(7)**	0.132 g	0.46 mmol	MW 284.35

Figure S5: Green metrics components detailed calculation for the asymmetric dimer **7** .

Copies of NMR Spectra

^{1}H and ^{13}C spectra of compound **3** (DMSO, 400 MHz, 101 MHz)

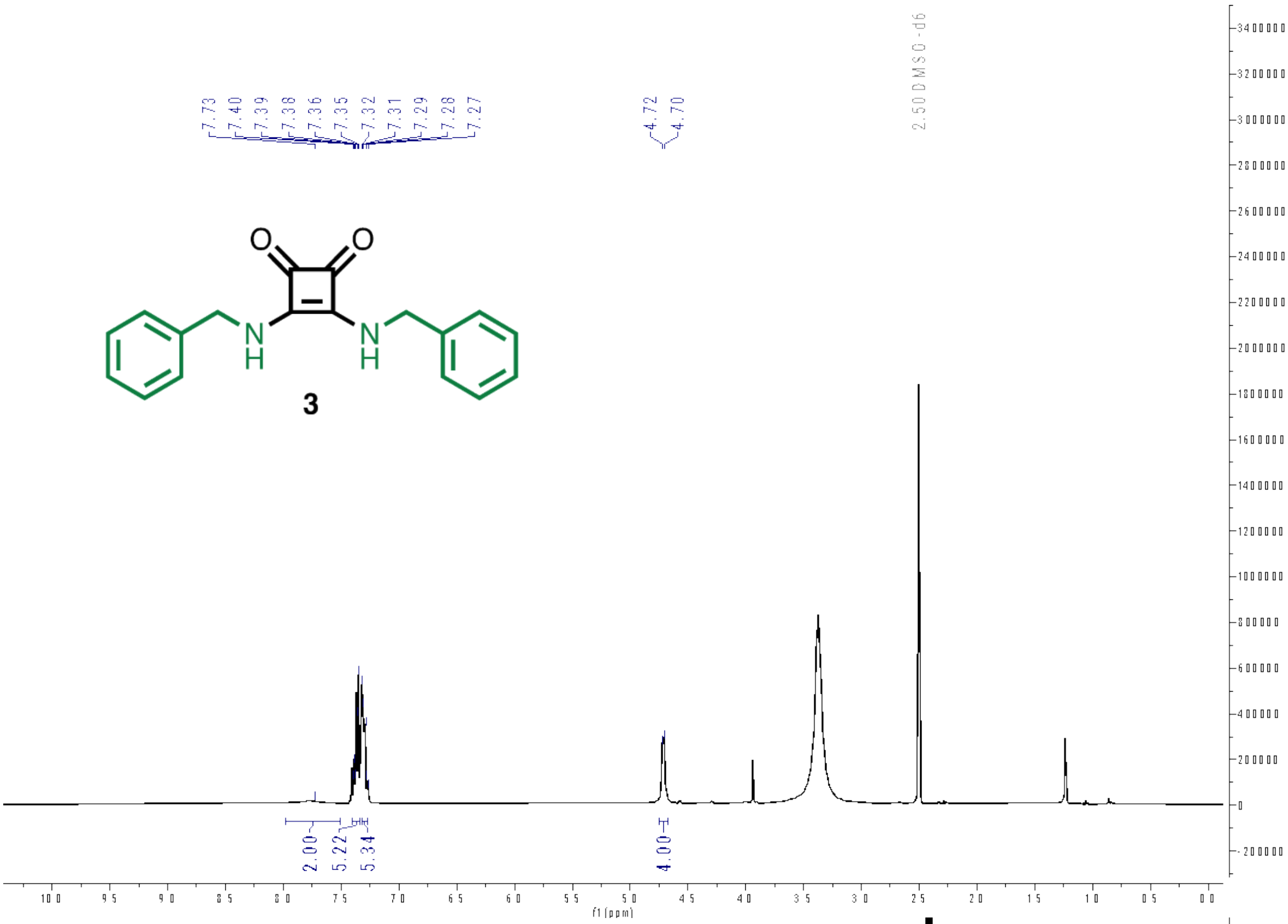

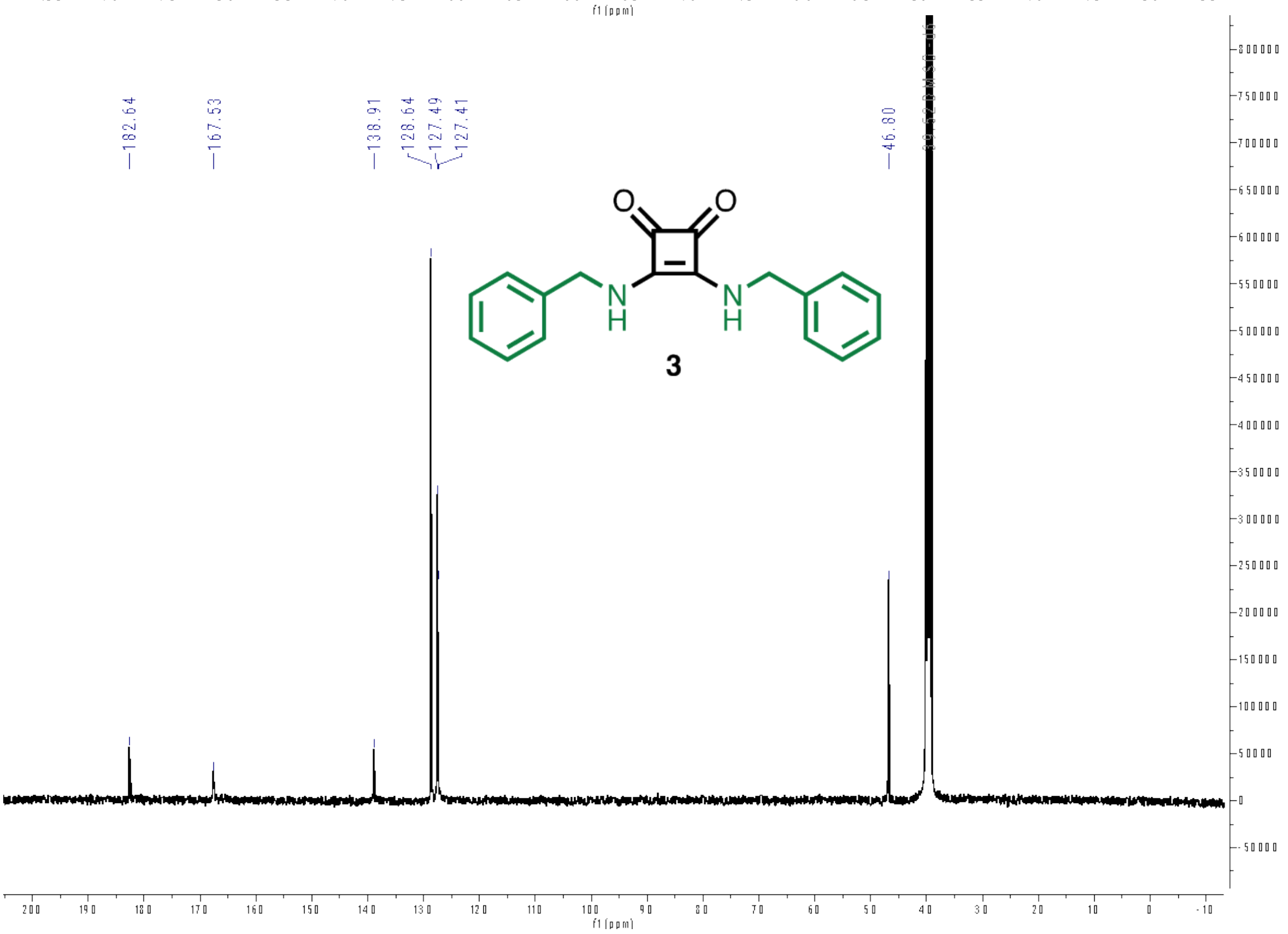

^{1}H and ^{13}C spectra of compound **5** (DMSO, 400 MHz, 101 MHz)

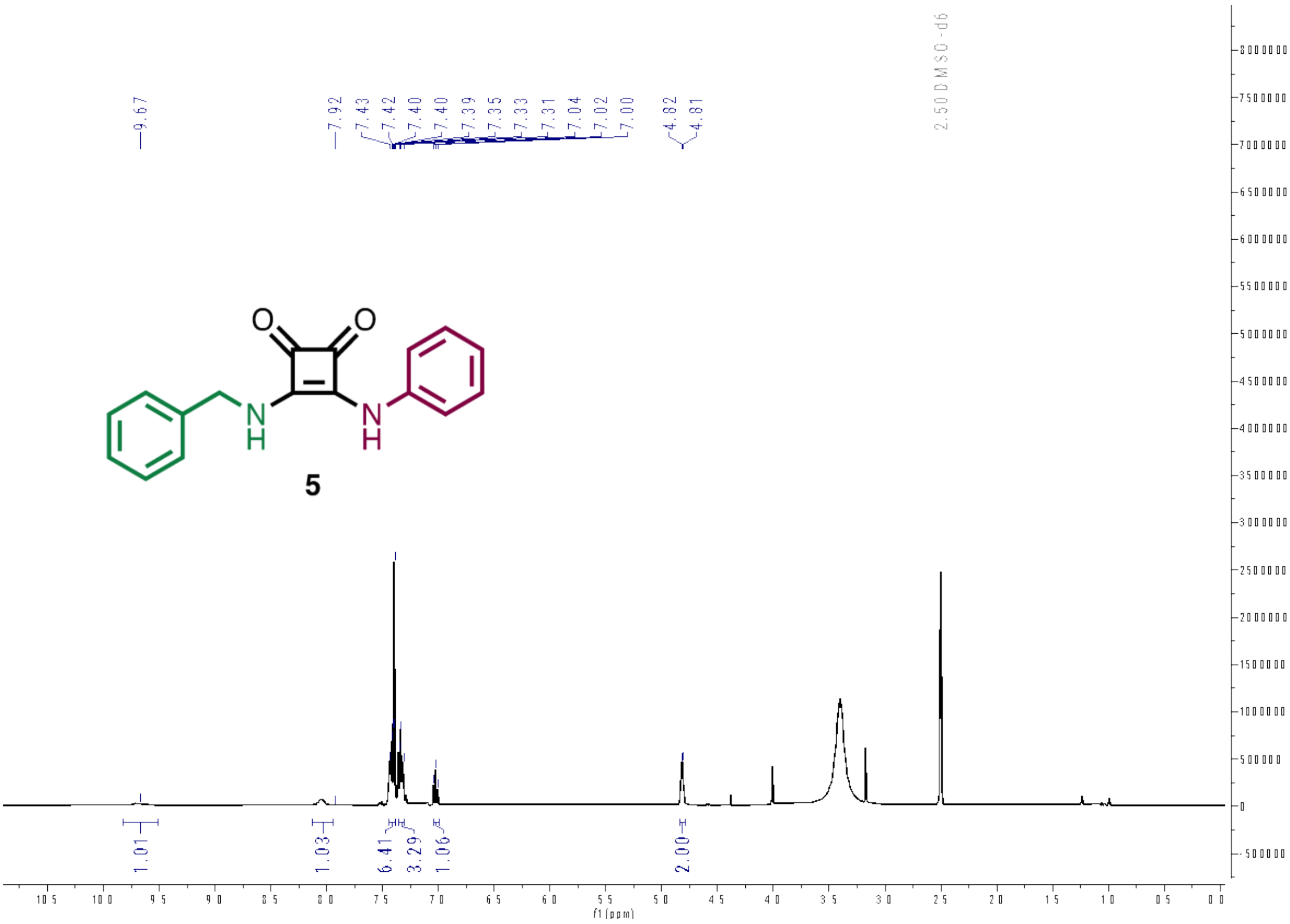

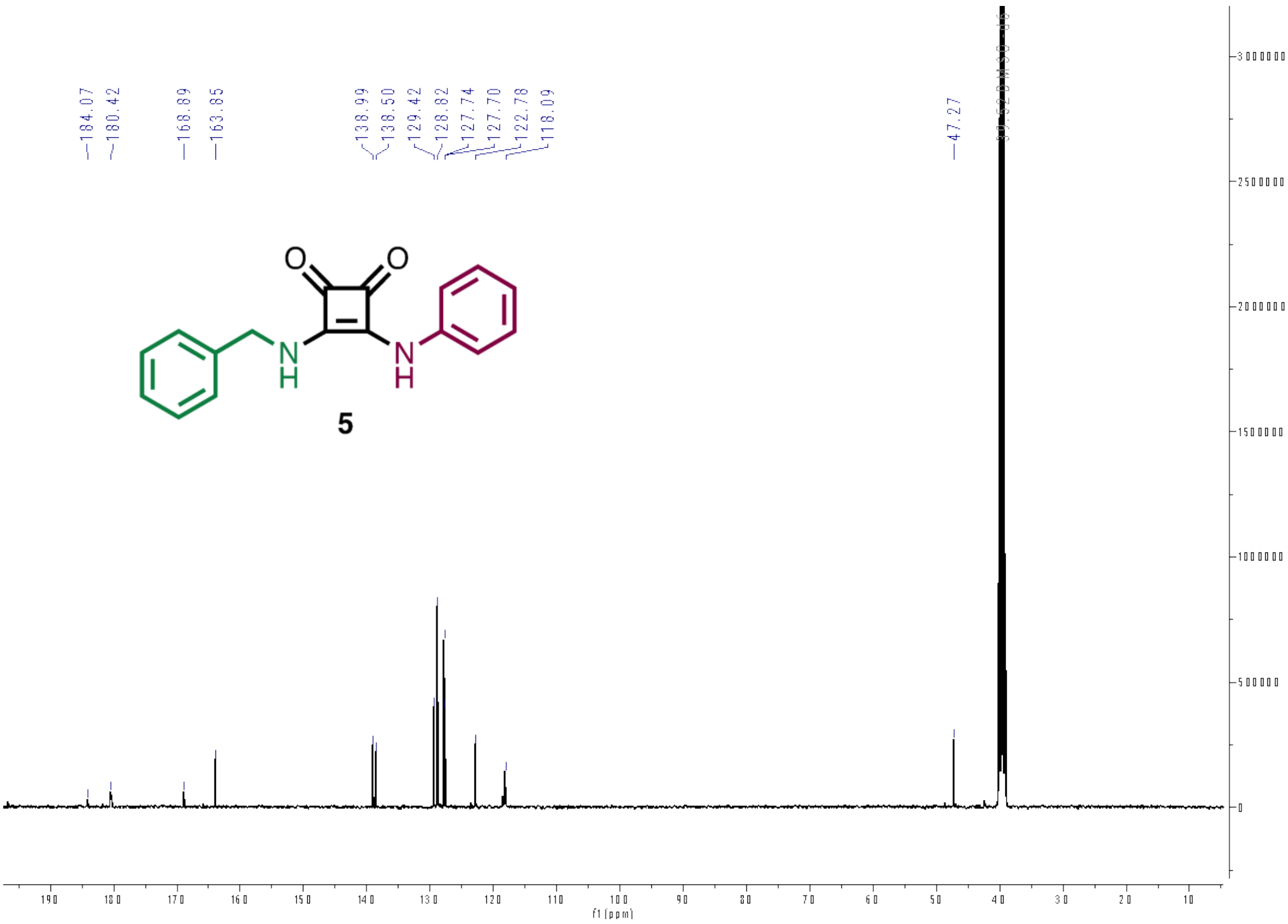

^{1}H and ^{13}C spectra of compound **7** (DMSO, 400 MHz, 101 MHz)

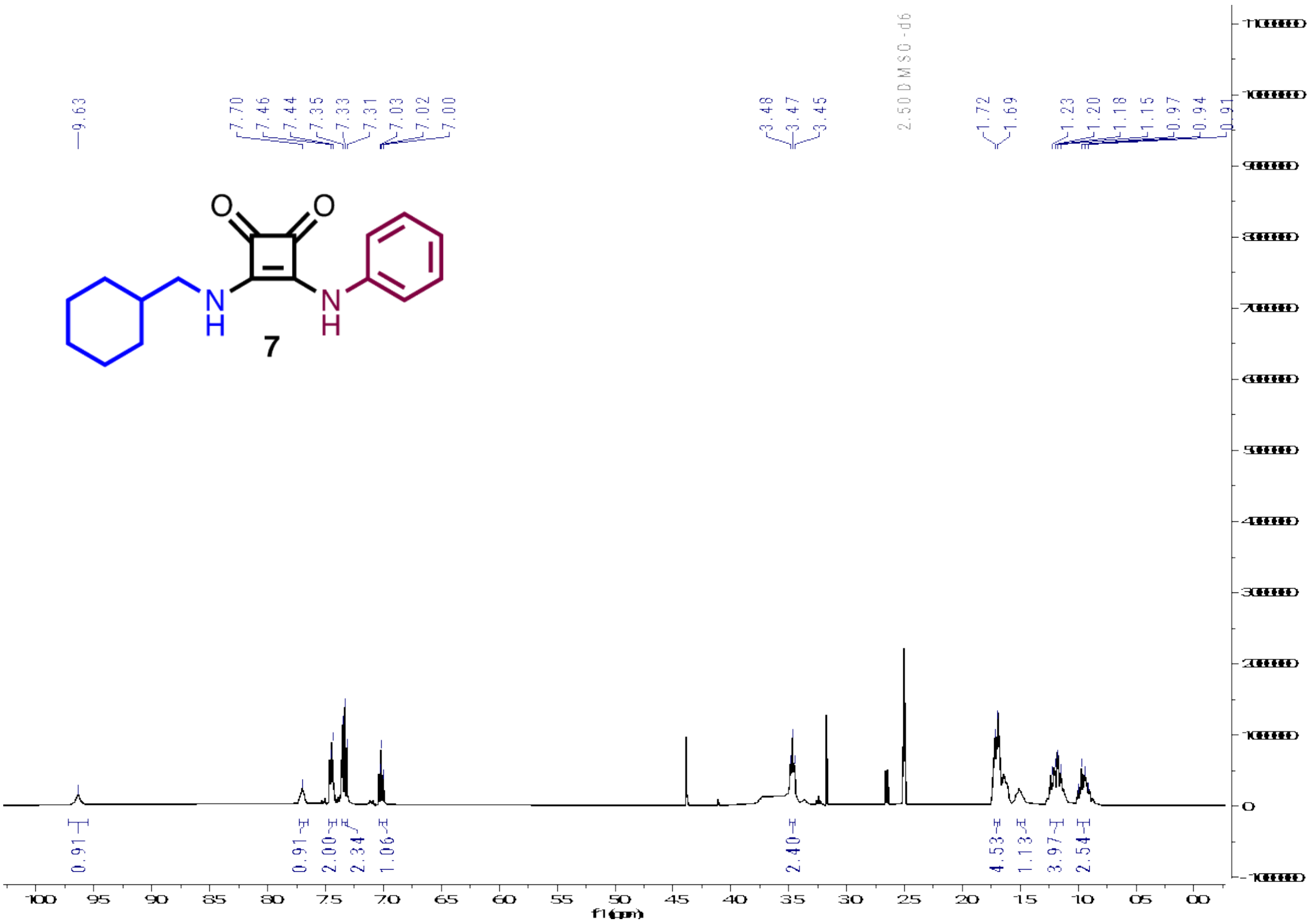

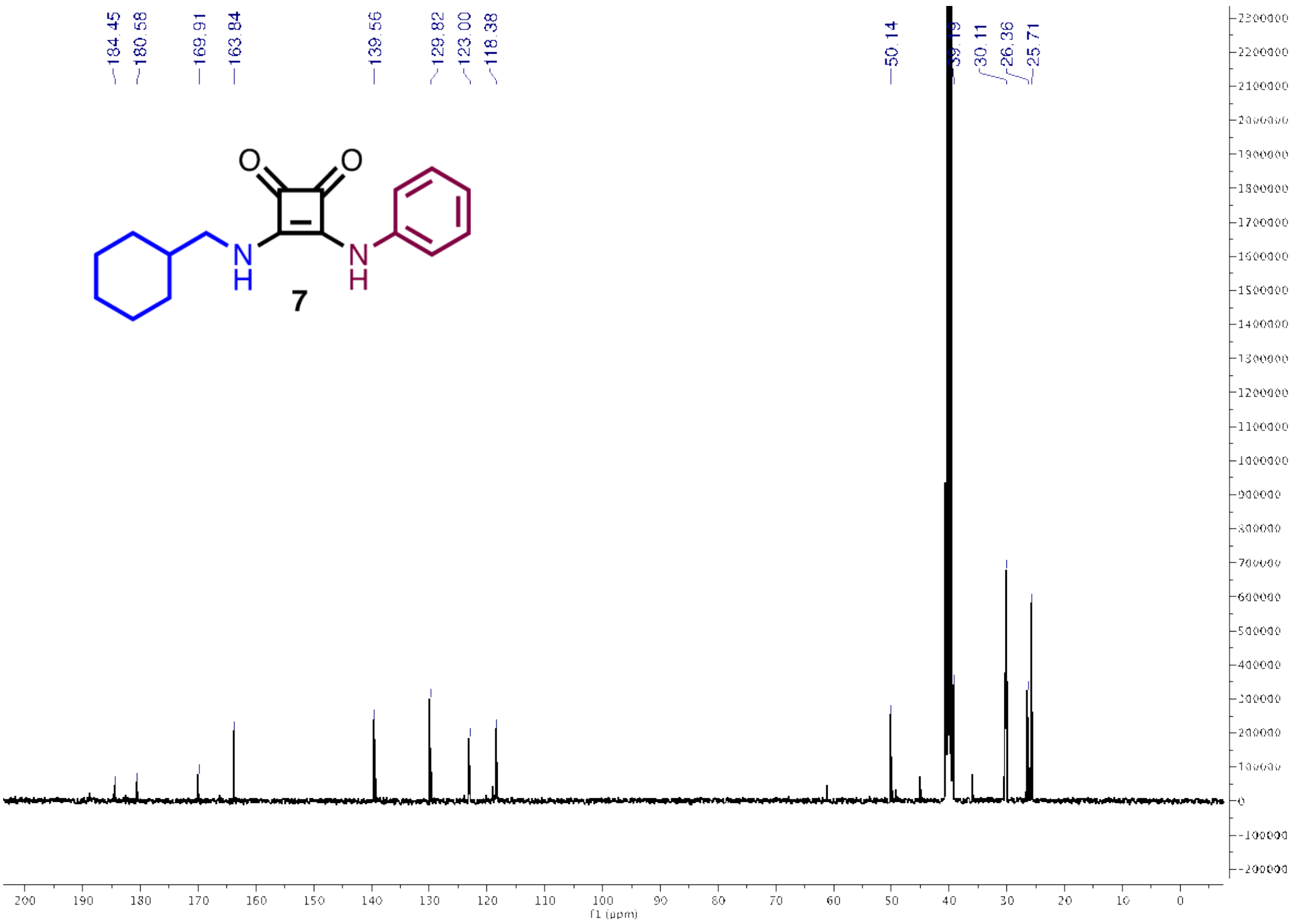

References

(1) Alfano, A. I.; Kalligosfyri, P. M.; Baia, V.; Brindisi, M.; Cinti, S. Streamlining Squaramide Synthesis Using a Sustainable and Versatile Paper-Based Platform. *Green Chemistry* **2025**, *27* (21), 5992–6001. https://doi.org/10.1039/D5GC00535C.
